# Greener approach for the isolation of oleanolic acid from *Nepeta leucophylla Benth*. Its derivatization and their molecular docking as antibacterial and antiviral agents

**DOI:** 10.1016/j.heliyon.2023.e18639

**Published:** 2023-07-25

**Authors:** Ajay Sharma, Deepika Kathuria, Bhaskor Kolita, Apurba Gohain, Ashoke Kumar Das, Garima Bhardwaj, Jesus Simal-Gandara

**Affiliations:** a Department of Chemistry, Sant Longowal Institute of Engineering and Technology, Sangrur, Longowal, Punjab, 148106, India; b Department of Chemistry, University Centre for Research and Development (UCRD), Chandigarh University, Gharuan, Punjab 140413, India; c Department of Botany, Jorhat Kendriya Mahavidylaya, Kenduguri, Jorhat, Assam, 785010, India; d Department of Chemistry, Assam University Silchar, Dorgakona, Silchar, Assam, 788011, India; e Department of Botany, Abhayapuri College, Abhayapuri, Srijangram, Assam, 783384, India; f University of Vigo, Nutrition and Bromatology Group, Analytical Chemistry and Food Science Department, Faculty of Science, E32004 Ourense, Spain

**Keywords:** *Nepeta leucophylla* benth., Medicinal plants, Oleanolic acid, Ultrasonication, In-silico antiviral potential, Antioxidant potential

## Abstract

In the present study bioactive methanolic extract along with chloroform and hexane extracts obtained from shade dried leaves of the Himalayan aromatic medicinal plant *Nepeta leucophylla* Benth. Were screened for the presence of triterpenoids, especially oleanolic acid (OA). Total three compounds oleanolic acid, squalene and linoleic methyl ester were isolated from methanol extract. The percentage yield of OA was 0.11%. Out of these three, OA is more bioactive and was further subjected to derivatization using greener Ultrasonication method. Total three derivatives (3-Acetyl oleanolic acid, 3-Phthaloyl oleanolic acid and 3-Oxo oleanolic acid) were synthesized with 91.16%, 93.98%, and 83.6% respectively. Further, the antioxidant potential of OA and its derivatives were evaluated using DPPH assay which suggested that the 3-Phthaloyl oleanolic acid exhibits highest antioxidant potential with 40.83 ± 1.14% inhibition. OA and its derivatives were screened *in-silico* antibacterial potential against three bacterial pathogens (*E-coli*, *M. tuberculosis* and *S. aureus*) and antiviral potential against Severe Acute Respiratory Syndrome Coronavirus (SARS-CoV-2), Human immunodeficiency virus (HIV) and H1N1 influenza virus. The *in-silico* results suggested that 3-phthaloyl oleanolic acid showed best H-bonding with FtsA (*Staphylococcus aureus*), enoyl acyl reductase (*E. coli*) and arabinosyl transferase (*Mycobactrium tuberculosis*). 3-Phthaloyl oleanolic acid also showed best H-Bond interactions with the target proteins hemagglutinin (H1N1) and reverse transcriptase (HIV), whereas, oleanolic acid exhibited the best interactions with RNA dependent RNA polymerase (SARS-CoV-2) and thus could be considered for further *in vitro* studies.

## Graphical abstract

**Figure:**
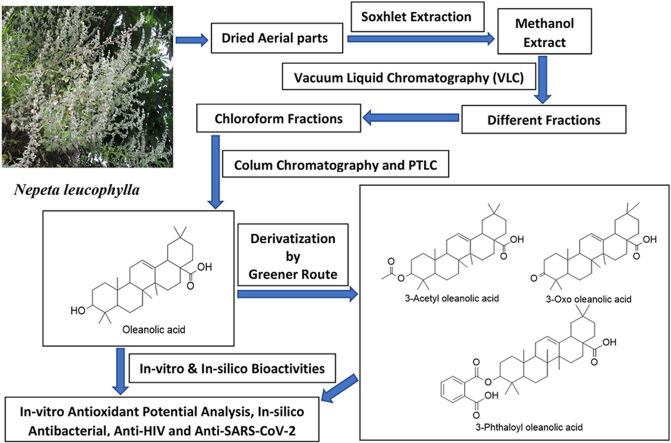

## 1 Introduction

Plant derived phytoconstituents or secondary metabolites (SMs) are well known for their diverse range of pharmacological properties from the ancient times. Owing to this, plant based phytoconstituents/SMs always attained continuous attention of the scientific world universally to obtain novel bioactive leads. These phytoconstituents or SMs are non-hazardous, eco-friendly, and are widely known for their innumerable applications for example nutraceuticals, agriculture, perfumery, personal hygiene, pharmaceutical and cosmeceutical [[1], [2], [3], [4], [5], [6]]. In order to counter the problem of resistance produced by the present antiviral and antibacterial drugs and owing to the toxic nature of most of synthetic antioxidants or preservatives, from precedent decade, the extensive search for plant based novel antibacterial, antiviral and antioxidants agents is underway [[7], [8], [9], [10], [11]]. Therefore, in the coming future there are innumerable projections to pursuit for the diverse array of natural bioactive antibacterial, antiviral and antioxidant agents of plant origin [[12], [13], [14]].

Naturally occurring pentacyclic triterpenoids are well known for their therapeutic and prophylactic potential against numerous diseases such as diabetes, hepatitis, multiple sclerosis, hypertension, ulcerative colitis, different cancers and metabolic disorders [[15], [16], [17], [18], [19], [20]]. Oleanolic acid (OA) is a pentacyclic triterpenoid secondary metabolite found in variety of aromatic and medicinal plants. The synthetic modification of naturally occurring OA at three reactive positions (the C (28)-COOH, C (12) = C (13) double bond and at C (3)-OH), led to the generation of new synthetic derivatives of OA. It has been reported that some of the synthetic derivative of OA showed better hepatoprotective and anti-inflammatory biological potential as compared to OA [21]. Recently, various *In-silico* studies also revealed that different plant based triterpenes have tremendous potential against SARS COV-2 Main Protease. Ursolic acid (isomer of OA) also reported to possess good *in-vitro* antiviral potential against rotavirus infections [22]. Therefore, due to broad pharmacological properties of triterpenes, OA and its derivatives can have potential to cure and manage several deteriorative and infectious diseases especially COVID-19 in coming future.

*Nepeta leucophylla* Benth. belongs to genus *Nepeta*, subfamily Nepetoideae and family Lamiaceae. *N. leucophylla* is a wild aromatic herb mainly found in Western Himalayan. It habitually grows on the open slopes of mountains [[23], [24], [25]]. Traditionally, the paste of *N. leucophylla* leaves is employed to treat malarial fever [26]. The Himalayan tribal communities also use *N. leucophylla* leaves to prepare herbal tea. Further, *N. leucophylla* essential oils and extracts have been reported to display antibacterial, antioxidant and antifungal activities. Furthermore, the analysis of phytoconstituents revealed the presence of catechin hydrate, chlorogenic acid, caffeic acid, coleon U 12-methyl ether, dihydroiridodial diacetate, syringic acid, iridodial β-monoenol acetate, iridodial dienol diacetate, myricetin, oleanolic, rutin trihydrate, squalene, ursolic acid, and vanillic acid, etc. in extracts of different parts of *N. leucophylla.* [3,24,25,27,28].

In our previous work on *N. leucophylla*, the effect of extraction methods and solvents on the bioactive composition especially polyphenolics were examined. The polyphenolics were investigated using RP-HPLC and the volatile constituents were evaluated with the help of headspace GC/MS and GC/MS. The antioxidant activity of various extracts from the different parts of plant were also assessed which suggested their high antioxidant potential as compared to positive control (quercetin and ascorbic acid) [3,24,29]. In continuance of our preceding work, in the current study, our focus is on the isolation of pure compound (mainly oleanolic acid) from the bioactive extract; to synthesize derivatives of isolated bioactive compound using greener synthetic techniques (Ultrasonication); finally, to assess the antioxidant potential (*in-vitro*). Further, the *in-silico* antibacterial and antiviral potential of all the compounds was evaluated.

## 2 Materials and methods

### 2.1 Chemicals and instruments

All reagents, chemicals and solvents used were of laboratory grade and used as it is without any purification. Sonicator with probe: CPX 500 model of Cole Parmer, USA (500 W, 20 kHz), Sonicator (bath): LMUC-4, Labman Scientific Instrument Pvt. Ltd. India (100 W, 40 kHz). Vacuum rotary evaporator (IKA RV-10, Germany: Buchi R-100, Switzerland), polarimeter (Anton Paar MCP 500), FT-IR (Bruker Tensor 27, Germany), UV–Visible analysis was carried out using Shimadzu Spectrophotometer 1800, GC-MS analysis was done using Shimadzu (GC-2010 Plus) GC system equipped with auto sampler, RTx-5Sil (30 m × 0.25 mm × 0.25 μm, Restesk USA) MS capillary column. MS-QP-2010 ultra (Shimadzu, Japan) was used for recording the mass spectra. Ultrashield 400 NMR spectrometer (Bruker Biospin, Rheinstetten, Germany) was used for recording the ^1^H and ^13^C NMR spectra at 400 MHz and 100 MHz respectively.

### 2.2 Collection of plant material and its processing

*N. leucophylla* aerial parts were collected from Manimhesh hills, near Hadsar - latitude 32.45 °N and longitude 76.61 °E; District - Chamba, Himachal Pradesh, India at an >2000 m altitude of in October 2016. To remove dust, debris and heavy soil particles the aerial parts were thoroughly washed with tap water. The aerial parts were segregated out into leaves (leaves + -flowers) and stem. The identification of plant under consideration was carried out by Prof. M.I.S. Saggoo, Prof. and Dean, Punjabi University, Patiala, India and deposited as PUN58876 in the Herbarium, Department of Botany, Punjabi University, Patiala. The leaves and flowers were dried in the shade at rt (24–32 °C) for the period of 30 days. After that the dried leaves and flowers were made into powder using an electric grinder which was stored in airtight polyethylene bags at a temperature less than 4 °C.

### 2.3 Isolation of different extracts

The previous studies on *N. leucophylla* already revealed that the extracts obtained by Soxhlet extraction method provided better results with respect to extraction yield, phytoconstituents composition and biological potential. So, in the current study Soxhlet extraction was utilized in order to get different extracts from the dried powdered leaves [3,24,29].

**Soxhlet extraction method (SEM):** The dried powder (80 gm in case of methanol extract, while 85 gm in case of chloroform and hexane extract) of *N. leucophylla* was extracted using various solvents with a varied polarity index such as hexane (PI = 0), chloroform (PI = 4.1), and methanol (PI = 5.1). Using 300 mL of the solvent, the extraction was performed at solvent boiling point with an extraction time of 36 h in every case. Further, the extracts were filtered (2 times) using Whatman filter paper (Grade no. 1). The extracts were dried using a rotary evaporator at temperature below 45 °C. Lastly, the % yields of extracts obtained were calculated. For further assessment, the extracts were stored at temperature below 4 °C.

### 2.4 Qualitative phytochemical analysis

Various reported qualitative tests were carried out to assess different photochemical classes such as alkaloids, carbohydrates, coumarins, flavonoids, glycosides, lignin, polyphenols, resins, steroids, saponins, and terpenoids (diterpenoids and triterpenoids), tannin, in the obtained extracts. For the indication of the presence or absence of any phytochemical class in the investigated extract, (+) or (−) symbol were used.

### 2.5 Isolation of phytochemical constituents

The previous studies on different parts of *N. leucophylla* along with present investigations revealed that the methanol extract obtained from powdered shade dried leaves provided better results with respect to extraction yield, phytoconstituents composition and biological potential [3]. Thus, herein, methanol extract was used to isolate pure triterpenoids (oleanolic acid).

#### 2.5.1 Vacuum liquid chromatography (VLC)

**Fig. 1:**
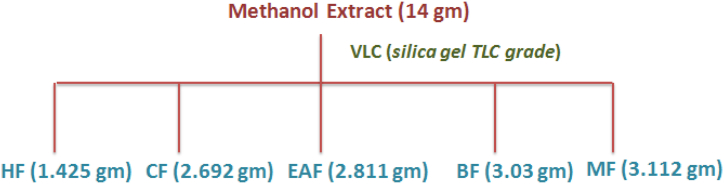
Isolation scheme of VLC.

14 gm of methanolic extract (obtained by SEM) was adsorbed on silica gel (200–400 mesh) which was loaded on a glass Buchner funnel (500 mL, VLC assembly) pre-packed with TLC grade silica gel. The elution was done starting from hexane, chloroform, ethyl acetate, butanol and methanol as follows. Total five fractions (Fig. 1) were obtained which include 1.425 gm of hexane fraction (HF), 2.693 gm of chloroform fraction (CF), 2.811 gm of ethyl acetate fraction (EAF), 3.03 gm of butanol fraction (BF) and 3.112 gm of methanol fraction (MF). Among the different fraction, only CF and HF give strong positive test (Liebermann - Burchard's and Salkowaski test) for the presence of triterpenoids and hence further taken for column chromatography to obtain pure triterpenoid compounds.

#### 2.5.2 Column chromatography (CC) of chloroform fraction (CF)

Hexane fraction (HF, 1.4 gm) was loaded on 60–120 mesh size silica gel and column with length 60 cm and dia. 40 mm. The gradient elution of hexane:ethyl acetate was utilized. The TLC was performed to analyze the collected fractions and the fractions with the same TLC were combined. In total, 21 fractions were gathered. Elution with 98% hexane gave first fraction. Further, 95 to 90% hexane was eluted, HF-2 was separated (a light orange color band). When the spot was analyzed using TLC, it revealed the presence of three minor and one major spot. The GC-MS analysis suggested squalene as a major component. Detail of rest, of compounds isolated given in the supplementary data file. Fig. S2 represents the isolation of HF.

#### 2.5.3 Column chromatography (CC) of chloroform extract obtained from the aerial part using SEM

Among the three extracts chloroform extract and methanol extracts gave strong positive test (Liebermann - Burchard's and Salkowaski test) for the presence of triterpenoids. Owing to this, only chloroform and methanol extracts were subjected to column chromatography analysis. Chloroform extract (CE, 5 gm) was loaded on silica gel (200–400 mesh) and the column was eluted hexane-ethyl acetate gradually to yield 242 fractions which were analyzed using TLC. Detail of rest, of compounds isolated given in the supplementary data file. Fig. S3 represents the isolation of CE. CF-2 and CE-3-1 (64 mg) were found to be the same which was revealed via TLC, melting point and mass spectrometry analysis and were identified to be oleanolic acid. Similarly, GC-MS analysis revealed that CF-1 and CE-2 were a mixture of two compounds.

### 2.6 GC-MS analysis

Table 1 represents the GC-MS conditions utilized to examine different fractions collected from chromatography. The recognition of phytochemicals was performed by comparing the obtained mass spectra with mass spectra library of databases (Wiley8 and NIST11) provided by the instrument. The quantification was carried out by calculating the peak area under the curve and results have been given in percentage.

**Table 1:** GC-MS conditions for the analysis of various fractions.

GC Condition			MS Conditions^a^	
Injection Temperature	280 °C		Ion Source Temperature	200 °C
Injection Mode	Split		Interface Temperature	280 °C
Column Flow (Helium)	1 mL/min		Solvent Cut Time	2.2 min
Split Ratio	5		Start *m*/*z*	40
Injection Volume	1 μL		End *m*/*z*	700
**Oven Temperature Program**			Start Time	2.5 min
**Rate (°C/min)**	**Temperature (°C)**	**Hold Time (min)**	End Time	78 min
	100	1		
4	250	5		
5	280	30		

a Mass spectra were recorded with EI mode at 70 eV.

### 2.7 Derivatization of oleanolic acid

Oleanolic acid is known to possess various biological potentials and thus chosen for derivatization.

#### 2.7.1 Acetylation with acetic anhydride

To a solution of 0.1 mM oleanolic acid in dry pyridine (2.5 mL), 1.3 mL acetic anhydride was added dropwise at ambient temperature and the mixture was sonicated for 15 min below 40 °C. The reaction progress was monitored using TLC. After completion of the reaction, the reaction mixture was poured on to ice cold water resulted in formation of white colored precipitates. The precipitated were filtered and washed thrice with water to obtain 45.4 mg white powder in 91.16% yield.

#### 2.7.2 Acetylation with phthalic anhydride

To a solution of 0.1 mM of oleanolic acid in 2.0 mL of dry pyridine, 0.4 mM phthalic anhydride was added slowly and the mixture was sonicated for 40 min below 40 °C. The reaction progress was monitored using TLC. After completion of the reaction, the reaction mixture was poured into ice cold water resulted in the formation of white colored precipitates. The precipitated were filtered and washed thrice with water to obtain 56.8 mg white powder in 93.98% yield.

#### 2.7.3 Oxidation of oleanolic acid

The solution of 0.2 mM oleanolic acid was prepared in 5 mL acetone at 0 °C. To the solution, slowly add 1 mL of freshly prepared Jone's reagent and the reaction was allowed to warm to ambient temperature, i.e. 25 °C. Further, the reaction mixture was sonicated below 25 °C for duration of 30 min. Next, the mixture was cool down to 0 °C followed by addition of isopropanol (10 mL). Again, the mixture was sonicated at room temperature for 10 min. Further, the mixture was filtrated to separate green color precipitates. The filtrate was concentrated using a rotary evaporator which was subjected to purification using column chromatography to obtain 75.9 mg white powder in 83.6% yield. The spectral data of all the isolated compounds and derivatives of oleanolic acids is presented in supplementary file.

### 2.8 Antioxidant potential

In this study, DPPH and TAC assays were performed for evaluation of the antioxidant activity of isolated compounds and synthesized derivatives. The assays were performed by taking ascorbic acid and quercetin dihydrate as a standard/positive control. The assays were performed in triplicate.

#### 2.8.1 DPPH free radical scavenging assay

Isolated compounds and their derivatives were subjected to access their DPPH free radical scavenging potential [24]. For the study, 0.2 mL of extract was mixed with a methanolic solution of 0.004% DPPH (3 mL) and was incubated in the dark at room temperature for the duration of 30 min (Fig. S32). The absorbance was measured by UV–Vis spectrophotometer at 517 nm. The percentage of the DPPH inhibition value is the mean of triplicate determinations. The DPPH scavenging activity (I %) was calculated using percentage inhibition formula i.e.

%inhibition = (Absorbance of Control- Absorbance of sample) / (Absorbance of Control) × 100

Where, Absorbance of control = the absorbance of DPPH + methanol, Absorbance of sample = the absorbance of DPPH + Plant extract/standard.

#### 2.8.2 Total antioxidant activity (TAC) assay

The TAC values of isolated compounds and their derivatives were assessed (Fig. S33) according to the phosphomolybdenum reducing procedure as explained by Sharma and Cannoo, (2016a). Total antioxidant activity was expressed as milligram ascorbic acid equivalents per gram sample (mg AAE/gm sample).

### 2.9 *In-silico* studies

The *in-silico* studies were carried out to investigate antibacterial and antiviral potential of the selected isolated compounds and their derivatives against the bacterial pathogens, *E-coli*, *Staphylococcus aureus*, *Mycobactrium tuberculosis* and viral pathogen, SARS-CoV-2, H1N1, HIV.

#### 2.9.1 Target's selection and molecular docking

To decipher antiviral and antibacterial properties of the selected isolated compounds and their derivatives, the following bacterial and viral proteins such as arabinosyl transferase (*Mycobactrium tuberculosis*), Enoyl acyl reductase (*E.coli*), FtsA (*Staphylococcus aureus*), hemagglutinin (H1N1), reverse transcriptase (HIV), RNA dependant RNA polymerase (SARS-CoV-2) are considered as main targets.

An arabinosyl transferase is a transferase enzyme which involves in the biogenesis of the mycobacterial cell wall and act as an important drug target [30]. The enoyl acyl carrier protein reductases commonly present in different *E. coli* species. It is mainly associated with fatty acid elongation and also acts as the precursor for biosynthesis of mycolic acid. So, enoyl acyl carrier protein reductase is one of the attractive targets for the development of drugs for the treatment of infections associated with *E. coli* diseases [31]. The FtsA is an essential protein of *Staphylococcus aureus* that plays a vital role in the cell division of bacteria by affixing FtsZ protein to the cytoplasmic membrane [32].

Hemagglutinin in H1N1 is a homotrimeric glycoprotein that have a globular stem and head along with three sialic acid binding sites to assist the virus binding to host cells, hence could be a significant drug target [33]. RNA dependant RNA polymerase (RdRp) is plays a crucial role in the replication of virus [34]. Reverse transcriptase of HIV catalyzes the biosynthesis of a double-stranded pro-viral DNA using the viral genomic RNA and is a beneficial target for different *anti*-HIV drugs [35].

Crystal structures of arabinosyl transferase (PDB ID 3PTY), Enoyl acyl reductase (PDB ID 1C14), FtsA (PDB ID 3WQU), hemagglutinin (PDB ID 1RUZ), reverse transcriptase (PDB ID 3V4I) and RNA dependent RNA polymerase (PDB ID 7BV2) were obtained from Protein Data Bank (PDB).

Molegro Virtual Docker (MVD, 6.0, Molegro-a CLC Bio Company, Denmark, Trial Version) was utilized for the preparation of proteins by the automatic preparation mode of MVD for performing molecular docking studies [36,37]. Any attached ligands, cofactors and water molecule found were eliminated during preparation. Grid-based cavity prediction algorithm of MVD was used for predicting the binding sites of the protein where grid resolution was kept at 0.8 Å covering the protein. Accessibility of the grid points were checked with placement of spheres of radius 1.4 Å. The van der Waals overlap analysis and random directions were chosen from the accessible grid point to survey if any inaccessible grid point hits on the way which was repeated for 16 different directions giving its accessibility if 12 or more of these lines hits an inaccessible volume. Volumes below 30.0 Å^3^ were discarded as irrelevant after connecting the neighboring grid points. Predicted cavities of each target protein were scored and ranked according to their volumes and the highest scoring one was selected. The properties of selected cavities are in Supplementary Table S1.

The structures were drawn with the help ChemDraw software (12.February 0, 1076, CambridgeSoft, UK) and energy was minimized in Chem3D Ultra software using MOPAC by fixing the Root Mean Square (RMS) gradient to 0.01 kcal/mol. All the compounds including reference drugs, isolated compounds and synthesized derivatives were docked in order to identify the active sites of the selected protein structures, so as to analyze their stability through interaction pattern evaluation in MVD. Moldock Score of MVD (grid based) was utilized as the scoring system with Moldock SE (Simplex Evolution) as the search algorithm in the virtual screening mode. Best poses of individual compound for particular protein were sorted out on the basis of their docking scores or Moldock scores, which is a statistical scoring function that converts interacting energy into numerical values [38].

### 2.10 Statistical analysis

DPPH and TAC assay results were expressed as mean ± standard deviation (SD) and performed in triplicates. Statistica and 7MS excel software were utilized for the evaluation of results.

## 3 Results

### 3.1 Percentage extractive yield (PEY)

Among solvents under study, methanol resulted in the highest extraction yield (17.66%) followed by chloroform (13.21%) and hexane (3.96%) owing to its high polarity and dielectric constant. This helps in the isolation of polar, medium polar and some non-polar compounds. The results of percentage extractive yield are given in Table 2.

**Table 2:** The results of percentage extractive yield of different isolated extracts.

Solvent	Yield (gm)/Percentage extractive Yield (%) (w/w)
Methanol	14.13/17.66
Chloroform	11.23/13.21
Hexane	3.37/3.96

The results of PEY revealed that the solvent polarity and percentage extraction yields are directly correlated. Similar correlation have also been reported previously in case of *Torilis leptophylla (pla*n*t)* and *Opuntia ficus-indica* (flowers) [39,40]. The qualitative phytochemical analysis suggested that, methanol extracted the maximum number of secondary metabolites followed by chloroform and hexane which might be attributed to its high dielectric constant and polar protic nature. A similar observation was also noted previously in case of *N. nepetella* (aerial parts), *N. praetervisa* (aerial parts) and *N. cataria* (leaves), respectively [41,42].

### 3.2 Isolation of phytochemical constituents

The order of phytochemical composition of different extract isolated by SEM from shade dried leaves was methanol > chloroform > hexane. The same order was also observed in case of total polyphenol content (methanol extracts:141.9 ± 3.86 > chloroform: 35.96 ± 1.08 > hexane extracts: 1.66 ± 0.46 mg of gallic acid equivalent/g dry plant extract) and total flavonoid content (methanol extracts: 394.48 ± 15.45 > chloroform: 90.92 ± 14.69> hexane extracts: 17.38 ± 2.62 mg rutin equivalent/g dry plant extract) [3]. Further, methanol and chloroform extracts showed strong positive test (Liebermann - Burchard's and Salkowaski test) for the presence of triterpenoids as compared to hexane extract. Therefore, methanol and chloroform extracts were subjected for chromatographic techniques to separate its phytoconstituents especially triterpenoids.

#### 3.2.1 VLC analysis of crude methanol extract

Five fractions i.e. 1.425 gm of hexane fraction (HF), 2.692 gm of chloroform fraction (CF), 2.811 gm of ethyl acetate fraction (EAF), 3.03 gm of butanol fraction (BF), and 3.112 gm of methanol fraction (MF) were attained by performing the VLC of crude methanol extract (obtained by SEM). Among them, HF and CF showed positive qualitative tests for triterpenoids and thus underwent for column chromatography to obtain the pure compounds. Further, EAF and BF fractions were complicated mixture of polar compounds which are difficult to isolate by column chromatography using silica gel as stationary phase. Further, the purification of isolated compounds was carried out using C_18_ cartridges, diaion HP 20 and sephadex LH 20 resin column.

#### 3.2.2 Isolation of pure compounds from CF

Three major components i.e. 5 mg of CF-1, 87 mg of CF-2, and 3.1 mg of CF-3 were obtained from CF after column chromatography followed by PTLC (details are given in SI). When visualize under long UV in UV–Visible chamber CF-1 showed only one fluorescent spot (blue color) on a TLC plate. However, the GC-MS analysis revealed that it was an inseparable mixture of phathalic acid ester and diterpene with pimarane scaffold (Fig. S1a). CF-2 (white solid, M.P. 308–310 °C) gave positive (+) Liebermann-Burchard test for triterpenoids. EI-MS spectrum of CF-2 exhibited molecular ion peak at *m*/*z* 456 and the base peak was observed at *m*/*z* 248. The library of mass spectrum suggested the peak could belong to oleanolic acid. The structure of the product was characterized using ^1^H NMR, ^13^C NMR, mass, and IR data (Figs. S5–S8 in supplementary data). ^1^H NMR, ^13^C NMR, mass and IR data confirm the presence of oleanolic acid in CF-2 which is consisted with the previous reports [43,44]. RP-HPLC analysis of CF-2 showed two peaks one major and one minor peak that may belong to ursolic acid (an isomer of oleanolic acid). The full spectroscopic characterization has been presented in supplementary file.

#### 3.2.3 Isolation of pure compounds from HF

**Fig. 2:**
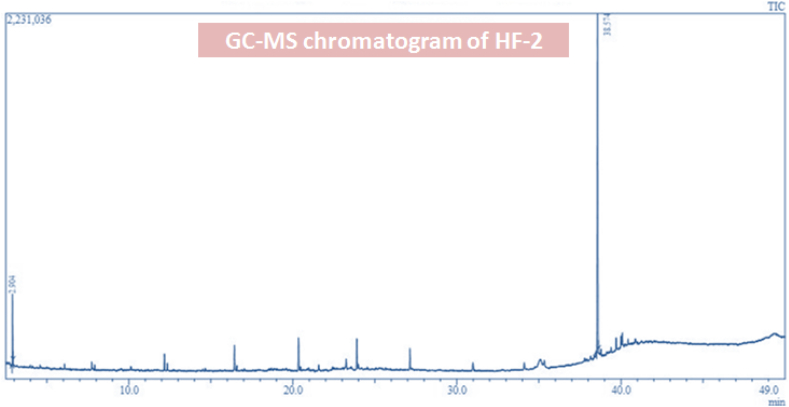
GC-MS chromatogram of HF-2 (the major peak was corresponding to squalene).

HF-2, HF-3, HF-5, HF-7 and HF-11 (Five major fractions) were obtained from the column chromatograph of hexane fraction (HF). The TLC of HF-2 suggested the existence of four compounds with one as a major component. Further, GC-MS analysis of HF-2 revealed the presence of squalene as a major component (92%) by matching the mass spectrum library (Fig. 2). HF-2 was further subjected to PTLC analysis which resulted in HF-2-1 where diaion HP 20 resin column and C_18_ cartridges was utilized for purification. The Liebermann-Burchard test was came out to positive (+) for HF-2-1. HF-2-1 EI-MS spectrum exhibited molecular ion peak at *m*/*z* 410 and the base peak was observed at *m*/*z* 69. The structure was characterized using ^1^H NMR, ^13^C NMR, mass and IR data (Figs. S9–S12). The data was consistent with previously published work [45]. The full characterization of the compound is provided in supplementary data file.

HF-3-1 was recognized as abieta-9(11), 8(14), 12-trien-12-ol (1.9 mg) (Fig. S29). Further, purification of HF-7-2 was done using column chromatography and PTLC. GC-MS profile of HF-7-2 showed one major peak and the library of mass spectrum (98.97%, library hit) suggested that the peak could belong to methyl ester of linolenic acid (Fig. S30). EI-MS spectrum of HF-7-2 exhibited M^+^ − 1 peak at *m*/*z* 291 and the base peak was observed at *m*/*z* 55. The structure was identified using ^1^H NMR, ^13^C NMR, mass and IR data (Figs. S13–S16). On the basis of ^1^H NMR, ^13^C NMR, mass and IR data, HF-7-2 was confirmed as methyl ester of linolenic acid. The data was further confirmed from the literature [46]. The full characterization of the compound is provided in supplementary data file.

#### 3.2.4 Isolation of pure compounds from chloroform extract

**Fig. 3:**
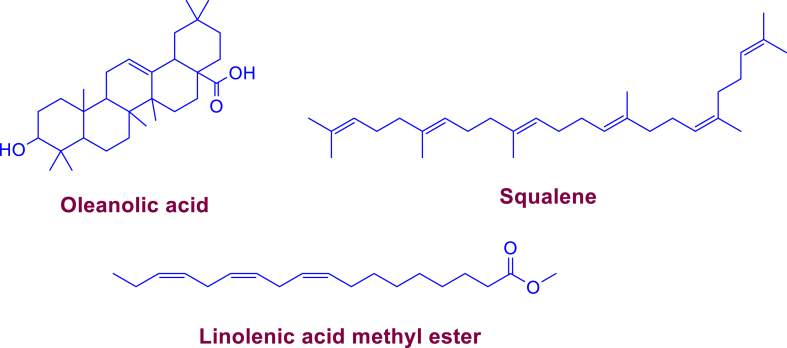
Structure of various isolated compounds a) oleanolic acid, b) squalene, and c) linolenic acid methyl ester form shade dried leaves of *N. leucophylla.*

Two pure phytochemicals were obtained from the column chromatography and PTLC of chloroform extract named CE-1-1 and CE-3-1. CE-1-1 is a light-yellow color viscous liquid. The analysis via TLC and GC-MS of CE-1-1 suggested its similarity with that of HF-2-1, squalene. Further, CE-3-1 was found to be identical (TLC, melting point and MS analysis) with CF-2 revealed that it was oleanolic acid. The isolation procedure of CE-1-1 and CE-3-1 is given in the supplementary information. Fig. 3 represents the structure of different isolated compounds from shade dried leaves of *N. leucophylla* (only those isolated in large amount)*.*

Overall, the present result revealed that chloroform fractions of crude methanol extract resulted in 1.09 mg of oleanolic acid whereas the crude chloroform extract provided 1.69 mg of oleanolic acid per gm of shade dried leaves powered. The result revealed that chloroform extract has higher content of oleanolic acid as compared to crude methanol extract. Further, the present yield of oleanolic acid in shade dried leaves of *N. leucophylla* is higher than that observed in apple skin, grapes peel, jujube pulp, lemon, olives pulp, *crataegus pinnatifida* leaves, *Eriobotrya japonica* flower and fruit peel, persimmon flesh and peel, *Sambucus nigra* bark, pears pulp and quince skin [15,17]. Furthermore, present method of isolation of oleanolic acid from methanol extract (VLC followed by column chromatography) of shade dried leaves of *N. leucophylla* is simple, less time consuming, cost-effective, economically viable, reproducible and more efficient in nature as compared to the previous reported methods in case of other plants. In present method, the percentage yield of oleanolic acid is about 0.11% in the case of methanol extract and about 0.17% in case of chloroform extract.

### 3.3 Derivatization of isolated bioactive compounds and evaluation of antioxidant potential of synthesized derivatives

**Fig. 4:**
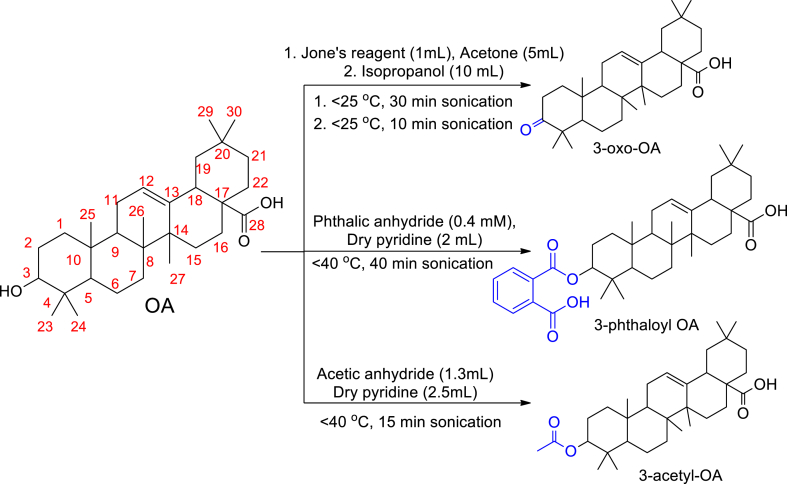
Scheme for the synthesis of oleanolic acid derivatives (OA-oleanolic acid).

The synthetic procedures for the derivatization are given in Fig. 4. The synthesized compounds were characterized using ^1^H NMR, ^13^C NMR, IR and mass spectrometry. The data was further confirmed from the literature [[47], [48], [49]]. The full characterization of the synthesized compounds is provided in supplementary data file (oleanolic acid - Figs. S16–S20; 3-Phthaloyl oleanolic acid - Figs. S21–S24; 3-oxo-oleanolic acid - Figs. S25–S28). The comparison of the conventional method and ultrasonication method used herein suggested that the ultrasonication method is the highly influential approach (Table 3).

**Table 3:** Comparison of current synthesis method (Ultrasonication) with previously reported methods for the synthesis of oleanolic acid derivatives.

Name of derivative	Ultrasonication % Yield (Time, temperature)	Reported (Conventional Method) % Yield (Time, temperature)	References
3- acetyl oleanolic acid	91.16% (15 min, <40 °C, sonication)	100% (24 h, rt, st) 96% (12 h, rt, st) 94.4% (1 h, reflux), 94.7% (24 h, rt, st) 98% (12 h, rt, st) 92% (12 h, rt, st)	(Gnoatto et al., 2008) (Bhandari et al., 2014) (Para et al., 2014) (Pattnaik et al., 2016) (Rali et al., 2016)
3-oxo oleanolic acid	83.6% (30 min, <25 °C, sonication)	80% (14 h, 0–15 °C, st) 51% (1 h at 0 °C, 3 h at rt, st)	(Bhandari et al., 2014) (Basir et al., 2014)
Phathalic acid derivative of oleanolic acid	93.98% (40 min, <40 °C, sonication)	93% (24 h, rt, st)	(Para et al., 2014)

rt: room temperature, st: stirring.

### 3.4 Antioxidant potential of isolated compounds and their derivatives

The antioxidant potential of isolated as well as synthesized compounds was evaluated using DPPH free radical and TAC assay (Table 4). Among the tested compounds the highest DPPH free radical scavenging potential was exhibited by 3-phthaloyal oleanolic acid. The order of antioxidant potential is as follows 3-phthaloyal oleanolic acid (40.83 ± 1.14%) > 3- acetyl oleanolic acid (35.43 ± 0.66%) > oleanolic acid (23.66 ± 0.16%) > 3-oxo oleanolic acid (15.87 ± 1.11%), squalene (13.04 ± 0.28%) > linolenic methyl ester (8.32 ± 0.36%). The higher DPPH radical scavenging potential of 3-phthaloyl oleanolic acid and 3- acetyl oleanolic acid as compared to oleanolic acid may be attributed to their better solubility [47] and higher tendency to release the proton (from COOH group phthaloyl moiety of 3-phthaloyl oleanolic acid and acetyl group of 3- acetyl oleanolic acid) as compared to oleanolic acid because anion generated after proton release are stabilized by phenomenon of resonance which is not possible in case of oleanolic acid. 3-oxo oleanolic acid (15.87 ± 1.11%) have lower DPPH radical scavenging potential which may be related to the concept that 3-oxo oleanolic acid have ketonic group at C (3), therefore there is less probability for release of proton as compared to oleanolic acid (23.66 ± 0.16%) which have alcoholic group at C (3).

**Table 4:** Results of antioxidant potential of isolated pure compounds from different extracts of *N. leucophylla* and their synthesized derivatives.

Compounds	DPPH (%)	TAC (mg AAE/gm DPE)
Oleanolic acid (CF-2)	23.66 ± 0.16	16.93 ± 1.47
Squalene (HF-2-1)	13.04 ± 0.28	5.23 ± 1.13
Linolenic acid methyl ester (HF-7-2)	8.32 ± 0.36	3.19 ± 1.65
3- acetyl oleanolic acid	35.43 ± 0.66	7.37 ± 0.13
3-phthaloyal oleanolic acid	40.83 ± 1.14	7.57 ± 0.27
3-oxo oleanolic acid	15.87 ± 1.11	14.58 ± 0.89
Ascorbic acid	58.42 ± 0.16	–
Quercetin	89.52 ± 0.89	488.95 ± 7.95

TAC - total antioxidant capacity and DPPH- 2,2-diphenyl-1-picrylhydrazyl radical scavenging assay. The results of TAC assay were expressed as mg AAE/gm DPE and that DPPH of as % Inhibition.

### 3.5 *In-silico* study

In the present studies, *in-silico* evaluations were done to observe antibacterial and antiviral properties of the isolated compounds and synthesized derivatives through the analysis of binding studies of the compounds with various target proteins of pathogenic bacteria viz. *Mycobactrium tuberculosis, E. coli, Staphylococcus aureus* and viruses viz. H1N1, SARS-CoV-2, HIV. The selected ligands were docked in the active sites of the targets in MVD. Molecular docking results were analyzed based on the docking scores [MolDock Score (kcal/mol)] and binding interactions.

The present antibacterial *in-silico* studies revealed that all the isolated compounds and synthesized derivative showed a better Moldock score than reference drug ethambutol and near to reference drug ceftriaxone with each target (Table 5). H-bond interactions of ethambutol and ceftriaxone are given in Figs. S34–S35, Table S1 respectively. Ligand squalene showed best Moldock score followed by 3-phthaloyl oleanolic acid, 3-acetyl oleanolic acid, linolenic acid methyl ester, 3-oxo oleanolic acid and oleanolic acid against arabinosyl transferase. Almost similar pattern is observed in case Enoyl acyl reductase and FtsA. *In-silico* antiviral studies revealed that 3-Phthaloyl oleanolic acid showed moderate binding with RNA dependent RNA polymerase of SARS-CoV-2 with MolDock score −118.28 kcal/mol, all other compounds showed poor binding compare to reference drug remdesivir (Table 6). Among the different ligand squalene showed poor binding with all the three viral targets.

**Table 5:** Docking result of the isolated compounds and synthesized derivatives against selected targets proteins of bacterial pathogen.

Arabinosyl transferase (*Mycobactrium tuberculosis*)		Enoyl acyl reductase (*E-coli)*		FtsA (*Staphylococcus aureus*),	
Ligand	MolDock Score (kcal/mol)	Ligand	MolDock Score (kcal/mol)	Ligand	MolDock Score (kcal/mol)
aCeftriaxone	−158.375	aCeftriaxone	−182.418	aCeftriaxone	−138.44
Squalene	−152.319	Squalene	−161.442	Squalene	−137.889
3-Phthaloyl oleanolic acid	−138.988	3-Phthaloyl oleanolic acid	−153.851	3-Phthaloyl oleanolic acid	−104.414
Linolenic acid methyl ester	−130.177	3-Acetyl oleanolic acid	−131.213	Linolenic acid methyl ester	−101.346
3-Acetyl oleanolic acid	−108.996	Linolenic acid methyl ester	−124.244	3-Oxo oleanolic acid	−93.4265
3-Oxo oleanolic acid	−106.358	Oleanolic acid	−117.776	3-Acetyl oleanolic acid	−90.0785
Oleanolic acid	−105.411	3-Oxo oleanolic acid	−117.605	Oleanolic acid	−82.716
aEthambutol	−93.6689	aEthambutol	−81.4913	aEthambutol	−77.6369

a Antibacterial positive control drug.

**Table 6:** Docking result of the isolated compounds and synthesized derivatives against selected target proteins of viral pathogen.

Hemagglutinin (H1N1)		Reverse transcriptase (HIV)		RNA-dependent RNA polymerase (SARS-CoV-2)	
Ligand	MolDock Score (kcal/mol)	Ligand	MolDock Score (kcal/mol)	Ligand	MolDock Score (kcal/mol)
a Remdesivir	−149.169	3-Phthaloyl oleanolic acid	−154.286	a Remdesivir	−139.44
aEmtricitabine	−130.452	a Remdesivir	−145.679	3-Phthaloyl oleanolic acid	−118.28
3-Phthaloyl oleanolic acid	−111.524	aEmtricitabine	−137.129	Linolenic acid methyl ester	−106.30
3-Acetyl oleanolic acid	−107.359	3-Acetyl oleanolic acid	−121.229	3-Oxo oleanolic acid	−98.30
Linolenic acid methyl ester	−98.0342	Oleanolic acid	−107.773	oleanolic acid	−94.24
3-Oxo oleanolic acid	−93.1446	aZanamivir	−104.119	aZanamivir	−94.15
aZanamivir	−91.3862	Linolenic acid methyl ester	−103.354	3-Acetyl oleanolic acid	−93.23
Squalene	−85.4776	3-Oxo oleanolic acid	−102.244	aEmtricitabine	−89.60
Oleanolic acid	−78.691	Squalene	−79.979	Squalene	−89.27

a Antiviral positive control drug.

**Fig. 5:**
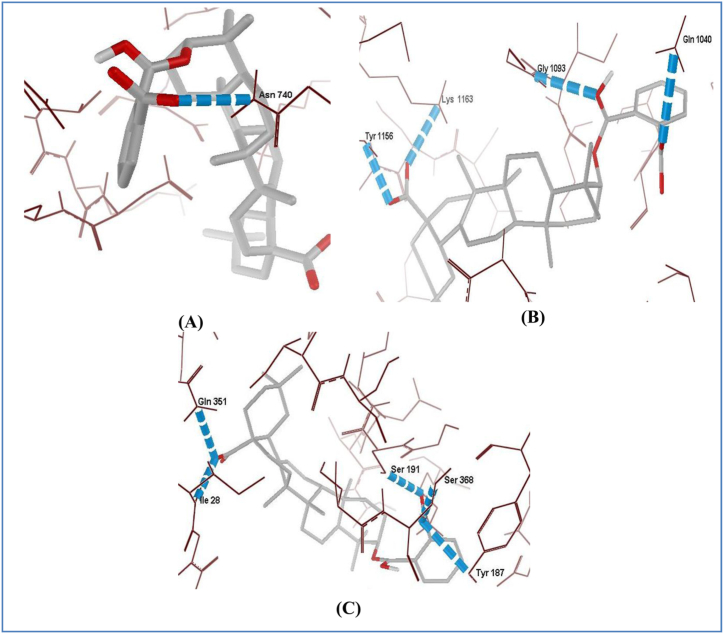
H-Bond pattern of 3-Phthaloyl oleanolic acid with interacting residues of A) arabinosyl transferase; B) enoyl acyl reductase; and C) FtsA.

To observe the ligand stabilizing properties, H-Bond interactions analysis was also carried out. H-bonds are weak and easily broken out, but play a major role in protein ligand interactions and also in stabilizing ligand protein interaction. H-Bond interaction in antibacterial targets, disclosed that overall ligand 3-phthaloyl oleanolic acid showed best H-bonding i.e. 1 H-Bond with arabinosyl transferase (*Mycobactrium tuberculosis*, Fig. 5A); 4 H-Bond with enoyl acyl reductase (*E. coli*, Fig. 5B); and 5 H-Bond with FtsA (*Staphylococcus aureus*, Fig. 5C), Other selected ligand showed 1-3 H-Bond with each antibacterial target (Figs. S35–S39). The molecular docking analysis suggests the antibacterial potential of the compounds under study.

**Fig. 6:**
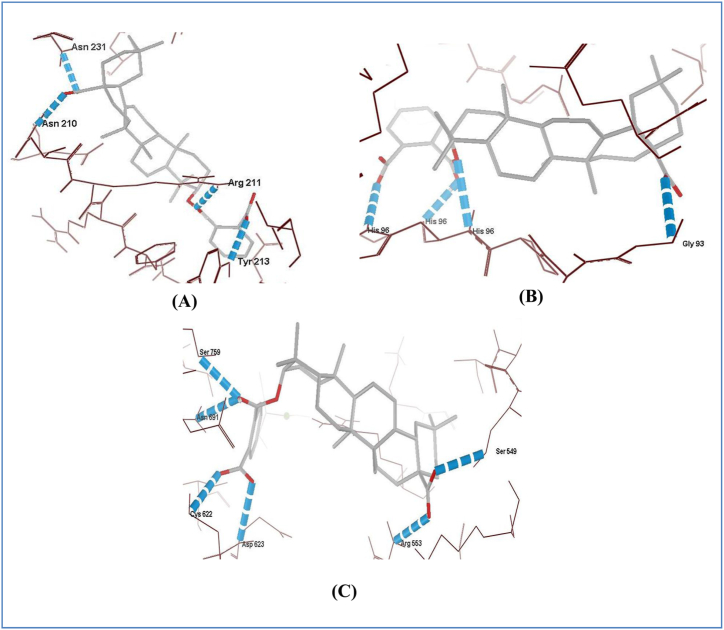
H-Bond pattern of 3-Phthaloyl oleanolic acid with interacting residues of A) hemagglutinin; B) reverse transcriptase; and C) RNA dependent RNA polymerase.

In case of binding against antiviral targets, the H-bonding interactions presented that overall 3-phthaloyl oleanolic acid showed best H-Bond interactions with the target proteins i.e. 4 H-Bond with hemagglutinin (H1N1, Fig. 6A), 4 H-Bond with reverse transcriptase (HIV, Fig. 6B); 6 H-Bond with RNA polymerase reverse transcriptase (SARS-CoV-2, Fig. 6C). The 3-phthaloyl oleanolic acid showed best H-Bond interactions of 6 H-Bond with RNA polymerase reverse transcriptase (SARS-CoV-2) amongst all the tested ligands which is comparable with tested reference drugs (Fig. S40). On the other hand, rest selected ligands showed 1-5 H-Bond with each antiviral protein target (Figs. S41–S46; Table S2).

## 4 Conclusion and future mandate

The present investigations revealed that among the different extracts isolated from leaves of *N. leucophylla*, methanol extract provided the best result of percentage extractive yield and qualitative phytochemical analysis. Upon VLC followed by column chromatography, methanol extracts provide three pure compound oleanolic acid, squalene and methyl ester of linolenic acid. The percentage yield of isolated oleanolic acid was about 011% in the case of methanol extract and about 0.17% in case of chloroform extracts. The present yield of oleanolic acid is much better than that reported previously in the case of *Allium sativum* (0.011%), *Arctium lappa* (0.00027%), *Betula utilis* (0.00043%), *Lotus corniculatus* var. viking (0.034%) and *Orthosiphon stamineus* (0.0004%). These results revealed that the present method (in case of methanol extract using VLC) is more economical and efficient than those used previously to isolate OA from different plants as stated above. Total three derivatives (3-acetyl oleanolic acid, 3-phthaloyl oleanolic acid and 3-oxo oleanolic acid) of OA were synthesized using greener ultrasonication technique. The ultrasonication method provided a better yield of synthesized derivative in a lesser time period as compared to previously used conventional methods for the synthesis these derivatives. These results revealed that ultrasonication method is more efficient and economical as compared to previously used conventional methods. Further, among all the isolated and synthesized compounds 3-phthaloyl oleanolic and oleanolic acid provided best results of *in-vitro* antioxidant activity, *in-silico* antibacterial and antiviral activities. Overall, the present *in-silico* studies predictions along with the reported antioxidant potential suggested that the OA and its derivatives have potential against studied pathogenic bacteria and viruses (hemagglutinin (H1N1), reverse transcriptase (HIV) and COVID-19 main protease (SARS-CoV-2) target proteins). The present investigations revealed the *in silico* bioactive potential of *N. leucophylla*. However, this plant has not been well explored for its complete biological potential. Therefore, the extracts and isolated compounds from *N. leucophylla* can be further evaluated for their *in-vivo* biological potential against different pathogenic bacteria, viruses and degenerative diseases. The outcomes of present investigations can help in the discovery and advancement of new potential drug or herbal formulations.

## Author contribution statement

Ajay Sharma: Conceived, designed and performed the experiments; analyzed and interpreted the data; and wrote the paper.

Deepika Kathuria: Analyzed and interpreted the data; wrote the paper.

Bhaskor Kolita: performed the experiments; analyzed and interpreted the data.

Apurba Gohain: performed the experiments; analyzed and interpreted the data.

Ashoke Kumar Das: performed the experiments; analyzed and interpreted the data.

Garima Bhardwaj: Analyzed and interpreted the data.

Jesus Simal-Gandara: Conceived and designed the experiments; contributed reagents, materials, analysis tools or data; Analyzed and interpreted the data; and wrote the paper.

## Data availability statement

Data included in article/supp. material/referenced in article.

## Additional information

Supplementary content related to this article has been publish online at [URL].

## Declaration of competing interest

The authors declare that they have no known competing financial interests or personal relationships that could have appeared to influence the work reported in this paper.
