# Supplementary material for: Greener approach for the isolation of oleanolic acid from Nepeta leucophylla Benth. Its derivatization and their molecular docking as antibacterial and antiviral agents
Source: Heliyon. 2023 Jul 25;9(8):e18639. doi: 10.1016/j.heliyon.2023.e18639 (PMC10407133; doi:10.1016/j.heliyon.2023.e18639)
Supplement: Multimedia component 1 [file mmc1.docx]

Greener Approach for the Isolation of Oleanolic Acid from *Nepeta Leucophylla Benth*. Its Derivatization and their Molecular Docking as Antibacterial and

Antiviral Agents

| **Table of Contents** |
| --- |
| S-0. Chemicals |
| S-1. Column chromatography (CC) of chloroform fraction (CF) |
| S-2. Column chromatography (CC) of hexane fraction (HF) |
| S-3. Column chromatography (CC) of chloroform extract obtained from aerial parts  using SEM |
| S-4. Spectral data of isolated compounds |
| S-5. Result and Discussion |
| S-6. In-silico Studies |
| References |

**S-0. Chemicals**

All solvents, chemicals and reagents used were of laboratory grade. All chromatographic purifications were carried out with silica gel #230-400, #60-120 and silica gel G (Spectrochem, Pvt. Ltd, Mumbai, India). Pre-coated TLC plates (silica gel coated with Kieselgel 60 -F254 and 0.2 mm thick, Merck, India) were used throughout the study. HPLC grade methanol (Sigma Aldrich, Pvt. Ltd, Mumbai, India), acetonitrile (Aldrich Co.), glacial acetic acid (Otto Chemie, Pvt. Ltd, Mumbai, India) were used for sample preparation and RP-HPLC-DAD analysis. The ultra pure millipore water was used throughout the research work, which was obtained from Millipore water kit (Millipore Direct Q 3). All other solvents, chemicals and reagents used in the present work were purchased from different agencies such as Merck, Pvt. Ltd, (Mumbai, India), Sigma Aldrich, Pvt. Ltd (Mumbai, India), Acros Organics (Thermo Fisher Scientific), Pvt. Ltd (Mumbai, India), Spectrochem, Pvt. Ltd (Mumbai, India), RANKEM (Avantor Performance Materials) Pvt. Ltd (Gurgaon, India), Loba Chemie Pvt. Ltd (Mumbai, India), Otto Chemie, Pvt. Ltd (Mumbai, India) and Sd fine chemicals, Pvt. Ltd (Mumbai, India).

**S-1. Column chromatography (CC) of chloroform fraction (CF)**

Similarly, fractions 55-57 (obtained when column eluted with 70 % hexane) were pooled together after TLC examination (hexane: acetone-6.5:3.5). The combined fractions upon preparative TLC yield single spot CF-3 (3.1 mg). The rest of all the fractions (6-33, 38-54 and 58-79) were dissimilar and with many spots in TLC. Further the amount of material in these fractions was lower and could not be isolated. The isolation scheme of CF is shown in Figure S1.

**Figure S1.** Isolation scheme of CF.


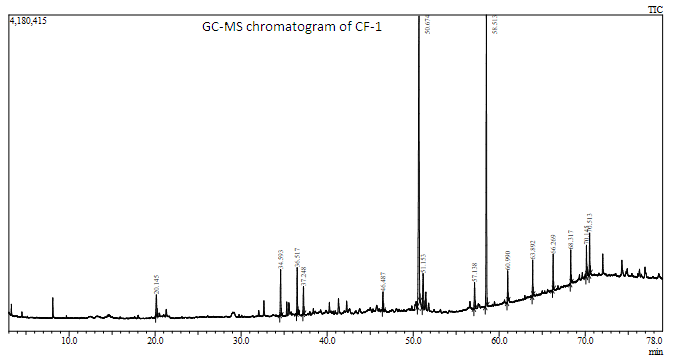


**Figure S1a.** GC-MS chromatogram of CF-1.

**S-2. Column chromatography (CC) of hexane fraction (HF)**

HF-2 was subjected to PTLC analysis and yielded HF-2-1 (23.2 mg) as single spot. The other sub-fractions of HF-2 were a mixture of compounds and the amount was also low. The TLC analysis of HF-3 showed the presence of one main spot, which was further purified with PTLC and yielded HF-3-1 (1.9 mg). GC-MS analysis of HF-3-1 showed one major peak (92%) corresponding to abieta-9(11), 8(14), 12-trien-12-ol. TLC analysis of HF-5 (8.3 mg) showed 3 major spots along with various minor spots. The GC-MS analysis of HF-5 showed the presence of menthyl acetate (44.49%), hexadecenoic acid (20.51%) and octadec-9-enoic acid (13.17%) as the key components. The compounds were difficult to separate due to small amount of fraction and small R_f_ difference in the compounds. HF-7 fraction was obtained, when column was eluted with hexane: ethyl acetate (80:20 - 75:25). TLC analysis revealed the presence of four spots. Further, PTLC of HF-7 gave HF-7-2 (single spot). GC-MS analysis of HF-7-2 showed one major peak which revealed the presence of methyl ester of linolenic acid (19.8 mg, 98.97%). TLC analysis of HF-11 showed single spot, the amount of HF-11 was very less (2.1 mg). GC-MS analysis of HF-11 showed one major peak with 87.1% abundance, but the compound could not be identified using standard GC-MS libraries. The other fractions (HF-1, HF-4, HF-6, HF-8 to HF-10 and HF-12 to HF-21) obtained from column chromatography of hexane fraction did not yield any pure compound and was the complex mixture of various compounds. Isolation scheme of HF is shown in Figure S2.

**Figure S2.** Isolation scheme of HF

**S-3. Column chromatography (CC) of chloroform extract obtained from aerial parts using SEM**

The light orange-colored fractions 16-24 were pooled together after TLC analysis. These pooled fractions were similar to the HF-2 fraction obtained from hexane fraction discussed above. TLC examination showed the presence of four spots. PTLC of pooled fractions (16-24) gave four sub fractions (CE-1-1 to CE-1-4) out of which CE-1-1 (6.7 mg) was pure and showed one major spot on TLC plate. The TLC and GC-MS analysis of CE-1-1 and HF-2-1 (obtained from hexane fraction of VLC) showed that both the compounds were same (squalene). Further, fractions 35-39 showed a prominent blue colored fluorescent spot on TLC plate when analyzed in UV-Vis chamber in short UV region along with other minor spots. TLC plates were developed in hexane: acetone (9:1) solvent system. The blue colored spot (CE-2, 9 mg) was separated with the help of preparative TLC (PTLC). The CE-2 was similar to the CF-1 that was obtained from chloroform fraction of methanol extract. Furthermore, the fractions 122-130 were pooled together after their TLC analysis. The TLC examination of fractions 122-130 revealed the presence of three main spots. The TLC plates were run in hexane: acetone (7:3) and developed with iodine and methanol sulfuric acid spray reagent. The pooled fractions (370 mg) were subjected to column chromatography on silica gel (200-400 mesh) using hexane and acetone as solvent, the column was monitor with the help of TLC. The column resulted in total 27 fractions and fractions 17-23 were same and pooled together. The TLC analysis showed one single spot named CE-3 (87 mg) with minor impurities. PTLC of CE-3 yielded pure CE-3-1 (64mg). Isolation scheme of CE is shown in Figure S3. The rest of fractions (1-15, 25-34, 40-121 and 131-242) obtained from column chromatography of chloroform extract were the complex mixture of various compounds. The amount of these fractions was also low and did not yield any pure compound.

The TLC, melting point and mass spectrometry analysis of CF-2 and CE-3-1 revealed that both the compounds were same. CF-2 and CE-3-1 were identified as oleanolic acid. Further, HPLC analysis of CE-3-1 showed that apart from oleanolic acid, it also contained some amount of its isomer ursolic acid (Figure S4). Similarly, CF-1 and CE-2 were a mixture of two compounds as revealed by GC-MS analysis.

**Figure S3.** Isolation scheme of chloroform extract obtained from aerial parts using SEM.


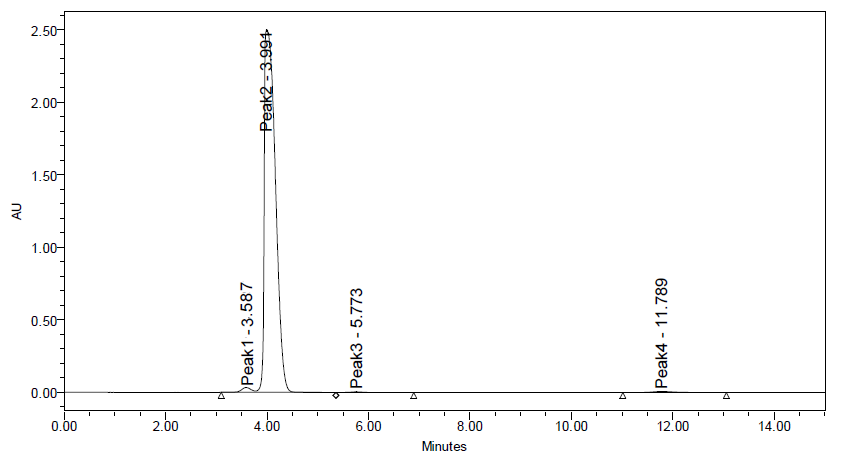


**Figure S4.** RP-HPLC chromatogram of CF-2.

**S-4. Spectral data of isolated compounds**

**S-4.1. CF-2 and CE-3-1: Oleanolic acid (OA) (Figure S5 – S8)**

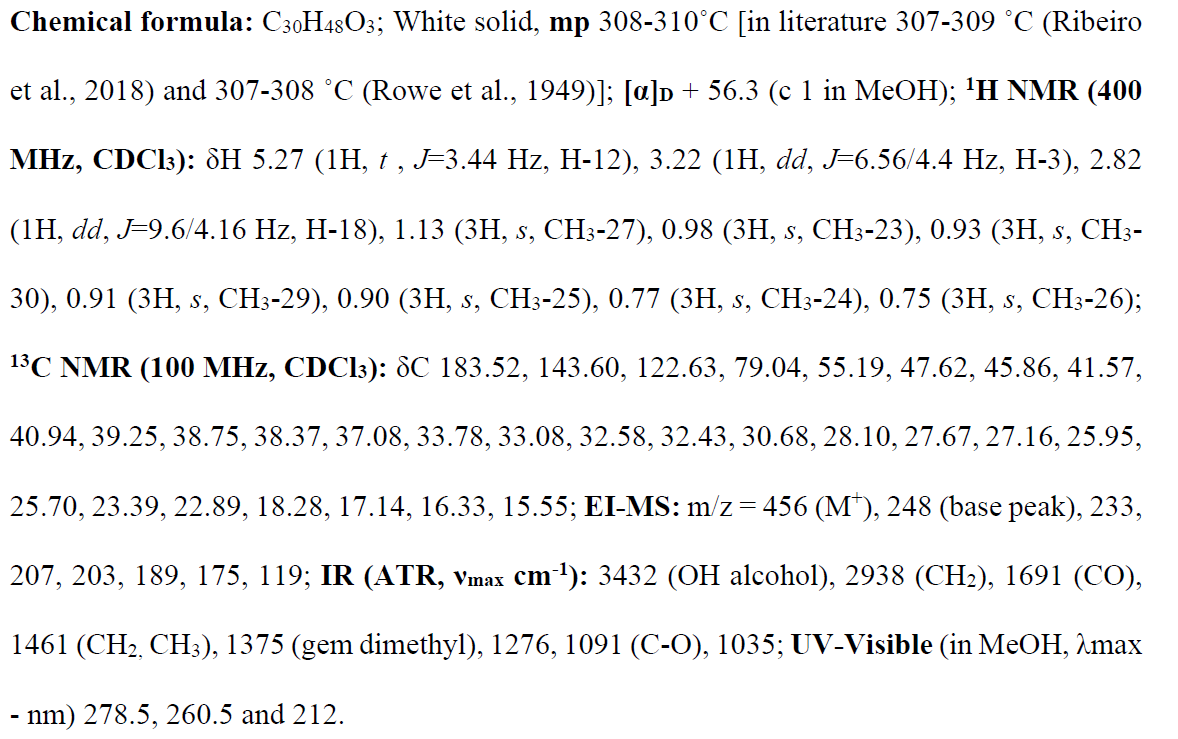


**
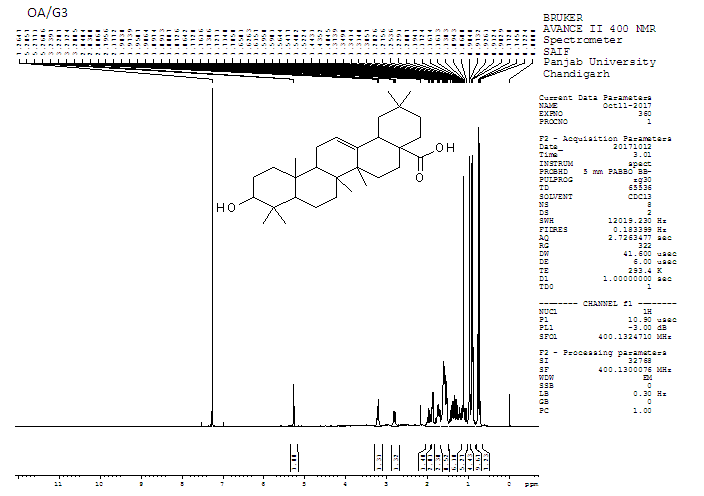
**

**Figure S5:** ^1^H NMR spectrum of oleanolic acid (CF-2)

**
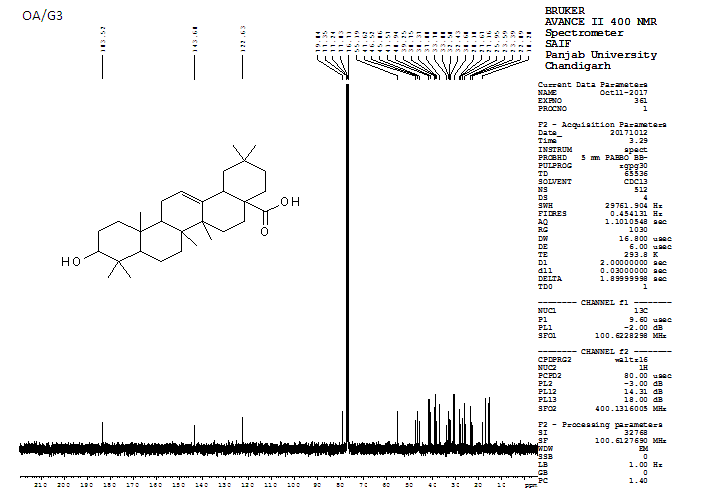
**

**Figure S6:** ^13^C NMR spectrum of oleanolic acid (CF-2)

**
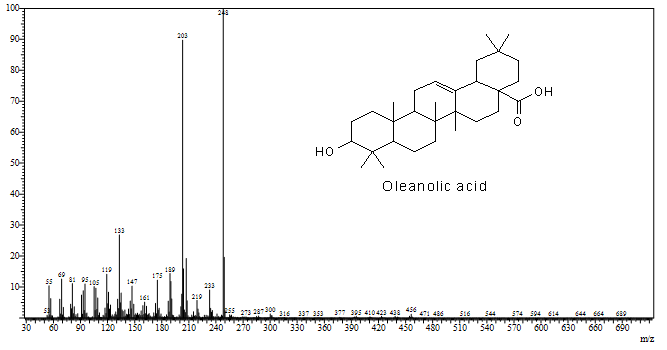
**

**Figure S7:** Mass spectrum of oleanolic acid (CF-2)

**
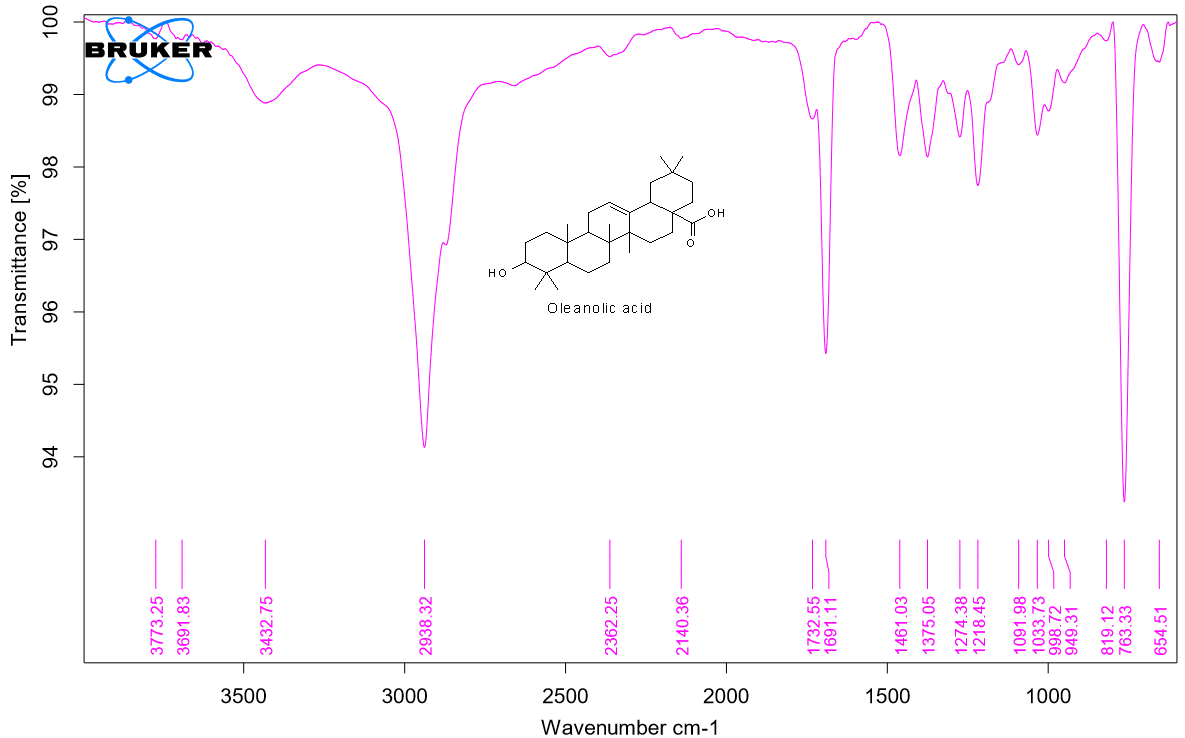
**

**Figure S8:** IR spectrum of oleanolic acid (CF-2)

**
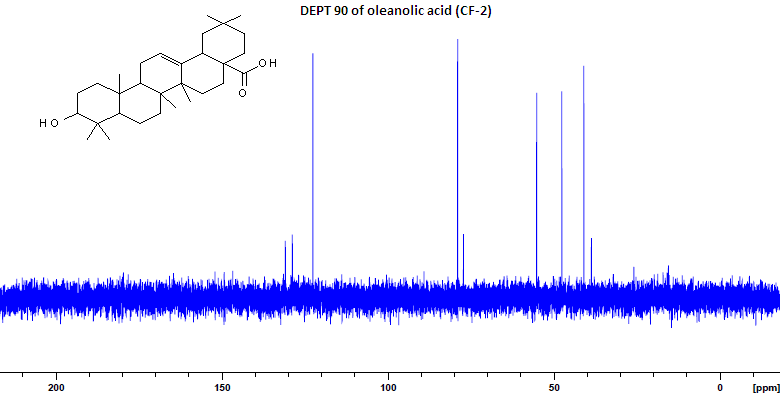
**

**Figure S8a:** Dept 90 of Crude CF-2


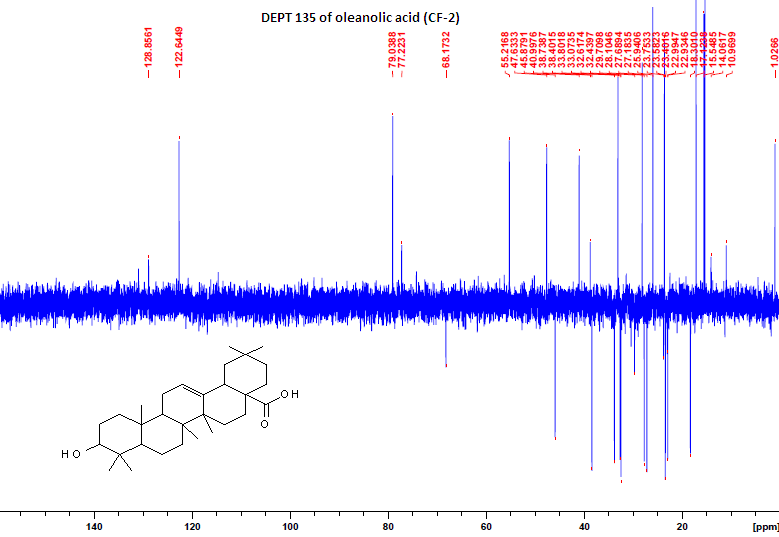


**Figure S8b:** Dept 135 of Crude CF-2

**S-4.2. HF-2-1 and CE-1-1: Squalene (SQ) (Figure S9 – S12)**

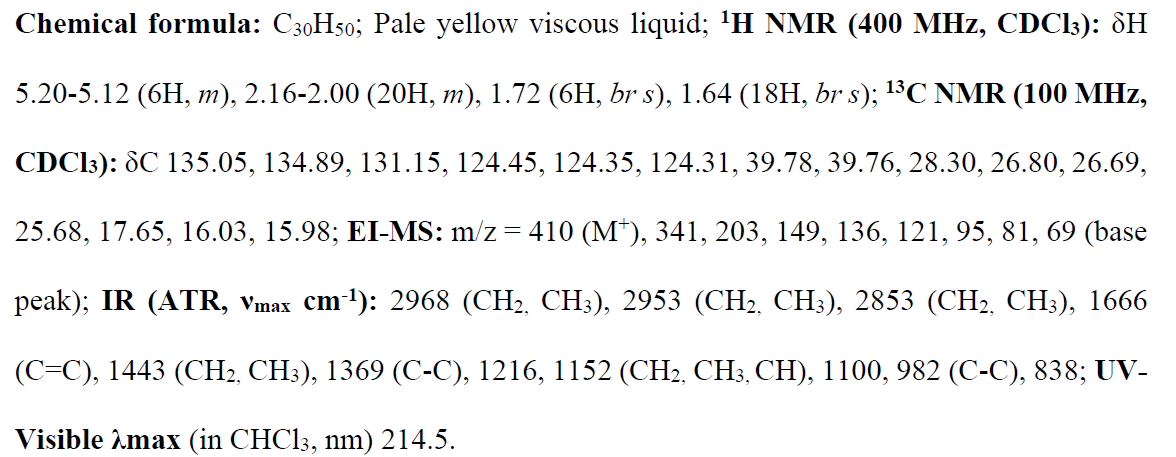


**
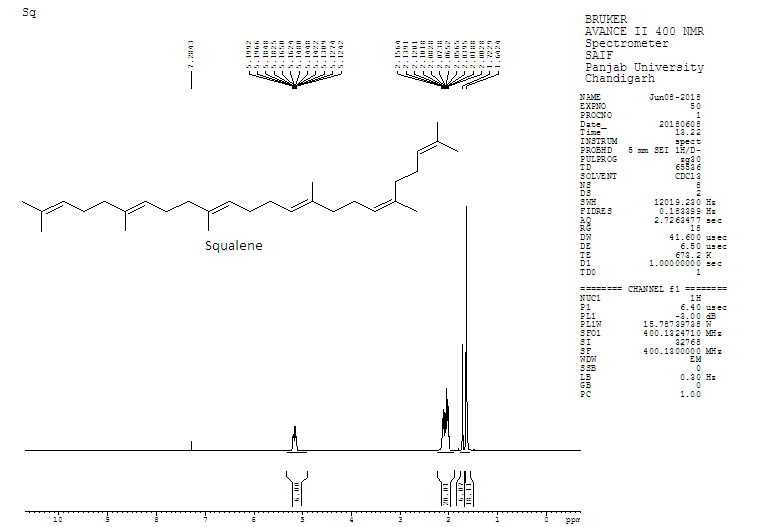
**

**Figure S9:** ^1^H NMR spectrum of squalene (HF-2-1)

**
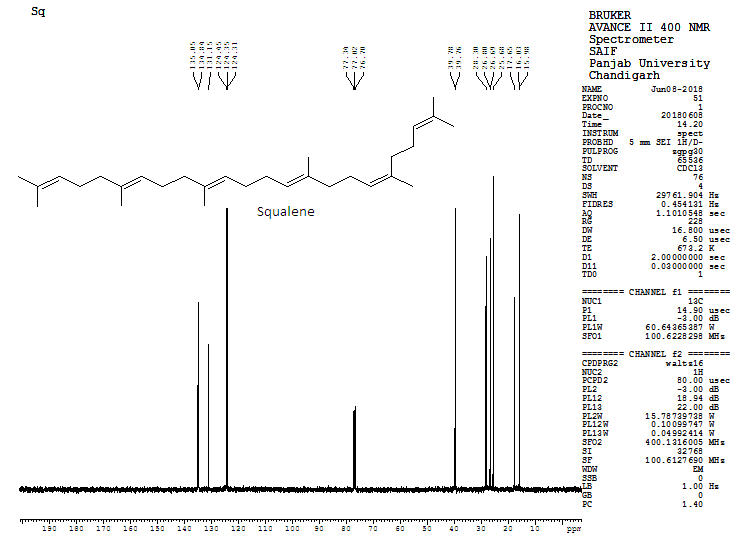
**

**Figure S10:** ^13^C NMR spectrum of squalene (HF-2-1)

**
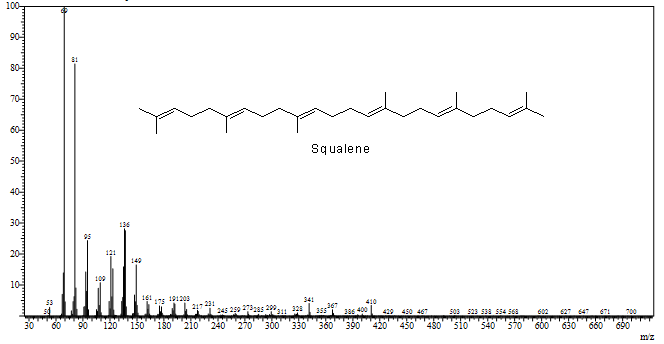
**

**Figure S11:** Mass spectrum of squalene (HF-2-1)

**
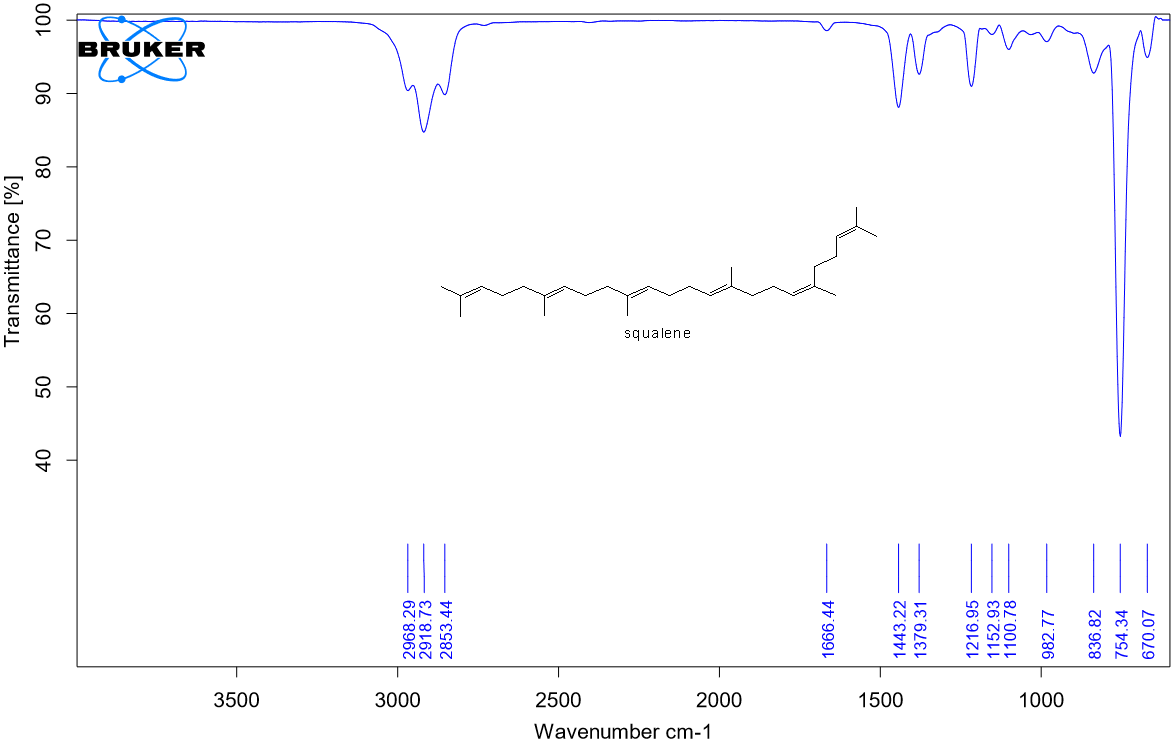
**

**Figure S12:** IR spectrum of squalene (HF-2-1)

**S-4.3. HF-7-2: Linolenic acid methyl ester (LAME) (Figure S13 – S16)**

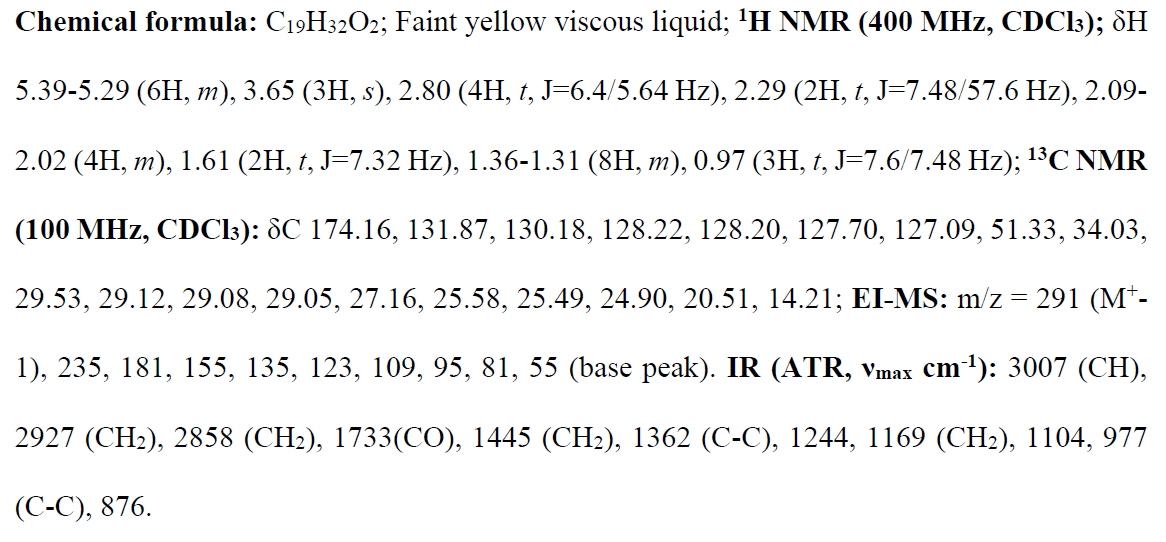


**
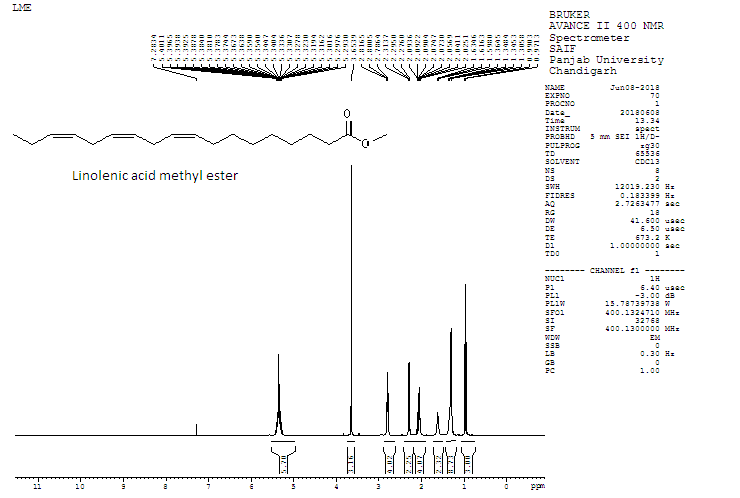
**

**Figure S13:** ^1^H NMR spectrum of linolenic acid methyl ester (HF-7-2)

**
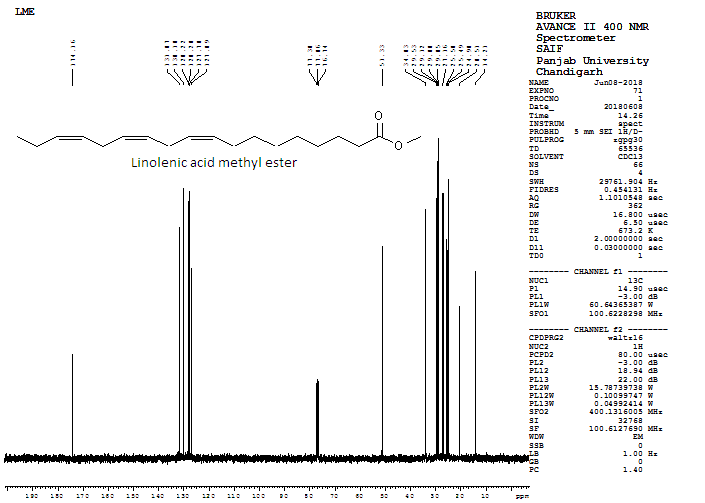
**

**Figure S14:** ^13^C NMR spectrum of linolenic acid methyl ester (HF-7-2)

**
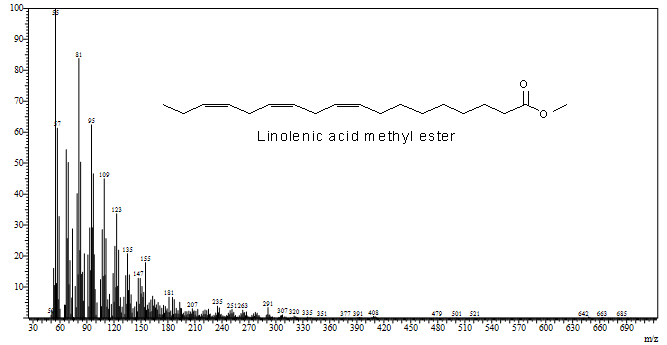
**

**Figure S15:** Mass spectrum of linolenic acid methyl ester (HF-7-2)

**
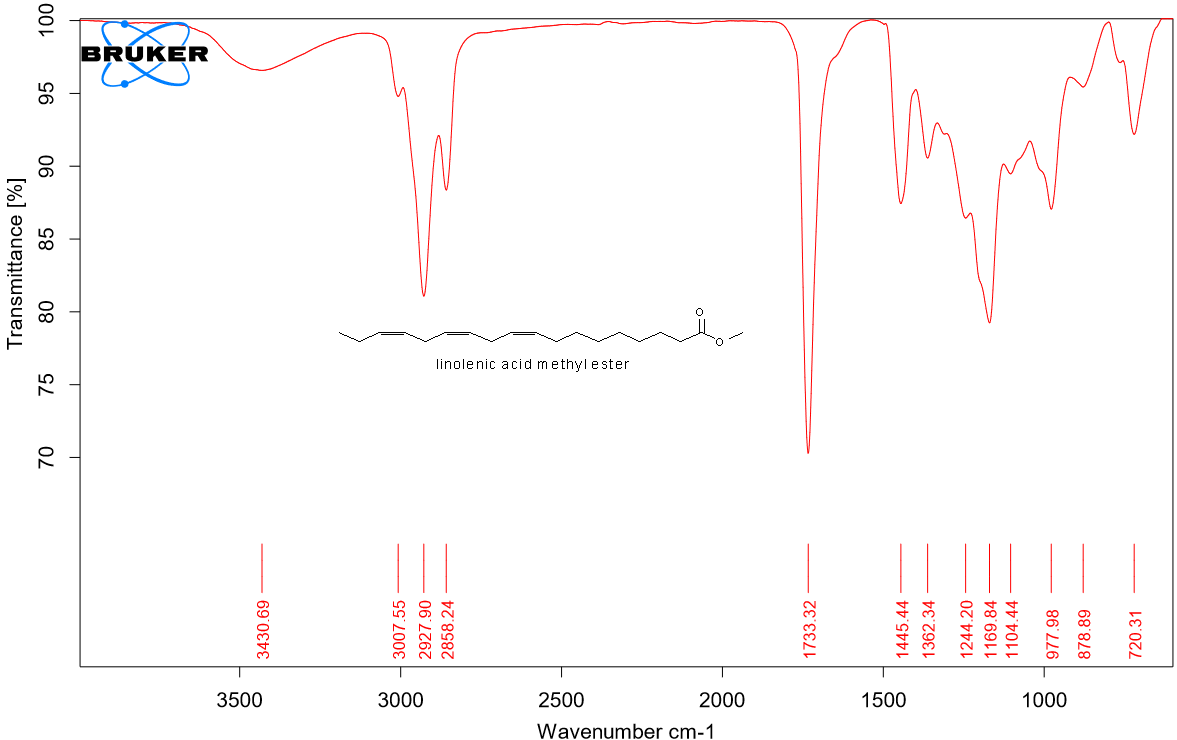
**

**Figure S16:** IR spectrum of linolenic acid methyl ester (HF-7-2)

**S-4.4. 3-Acetyl oleanolic acid (AOA) (Figure S17 – S20)**

**Reactants:** Oleanolic acid and acetic anhydride

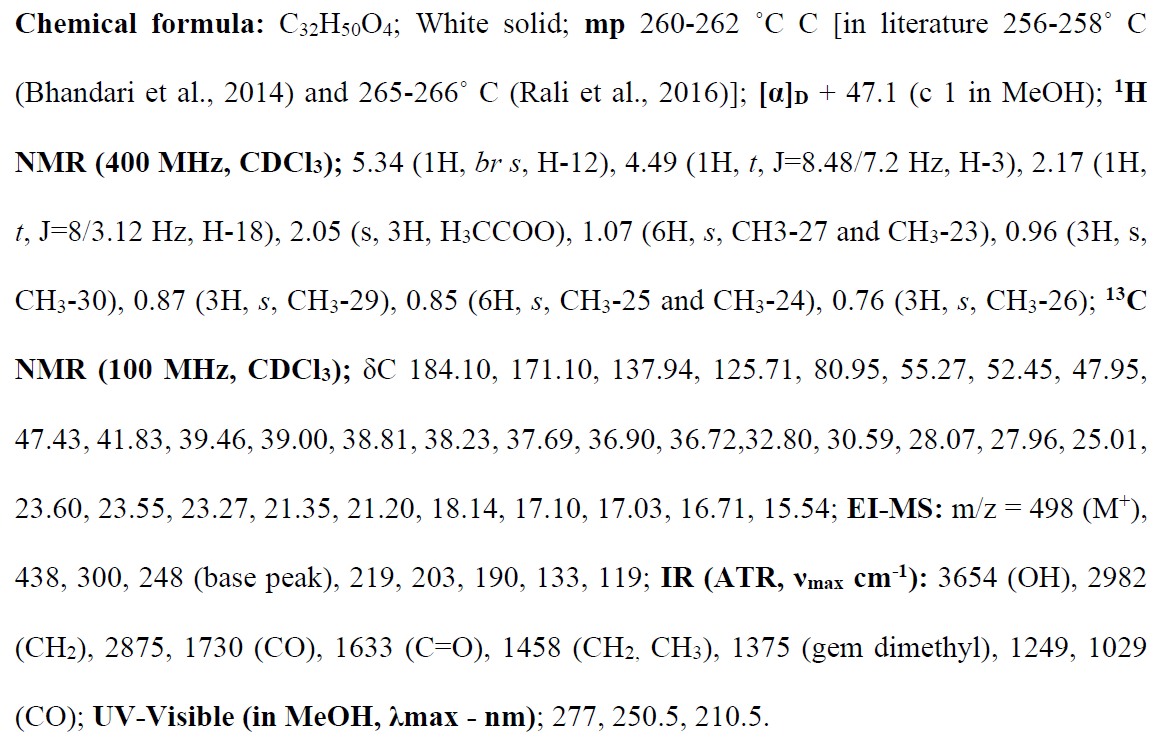


**
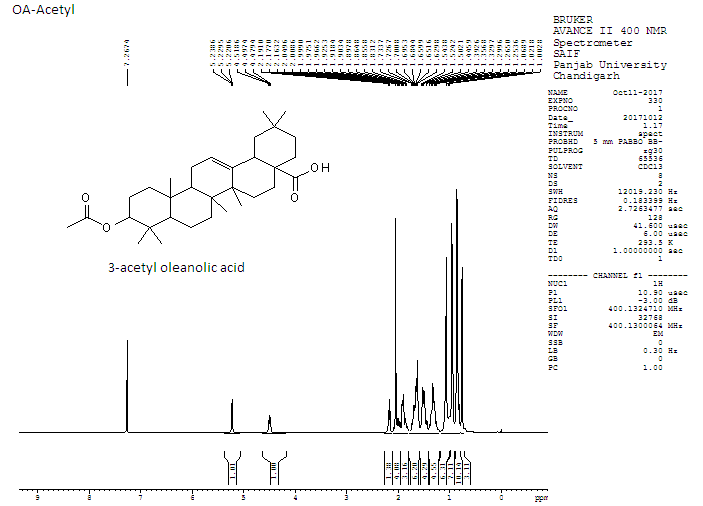
**

**Figure S17:** ^1^H NMR spectrum of 3-acetyl oleanolic acid

**
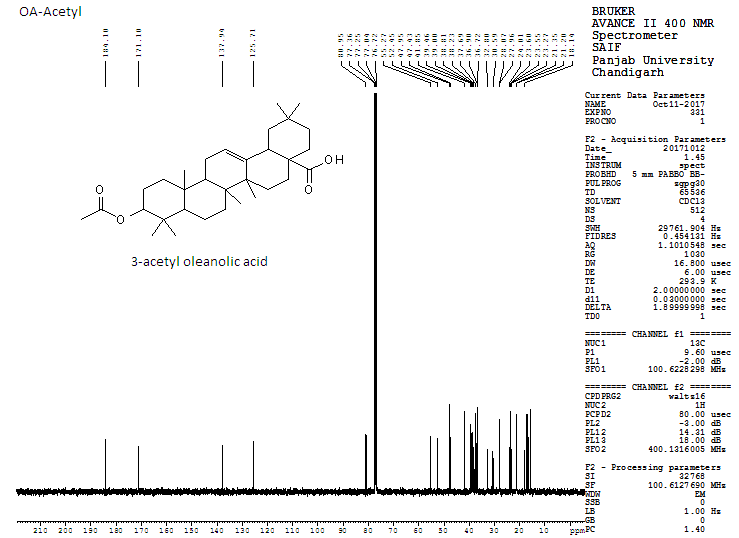
**

**Figure S18:** ^13^C NMR spectrum of 3-acetyl oleanolic acid

**
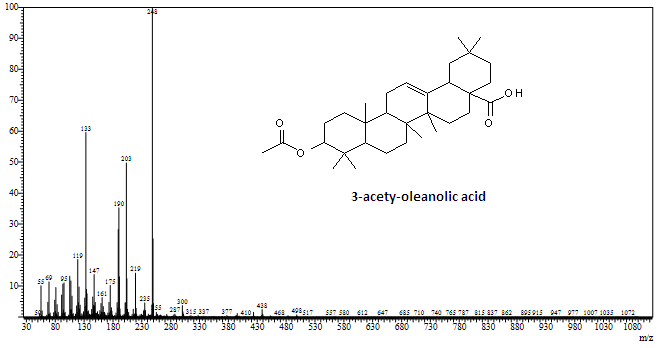
**

**Figure S19:** Mass spectrum of 3-acetyl oleanolic acid

**
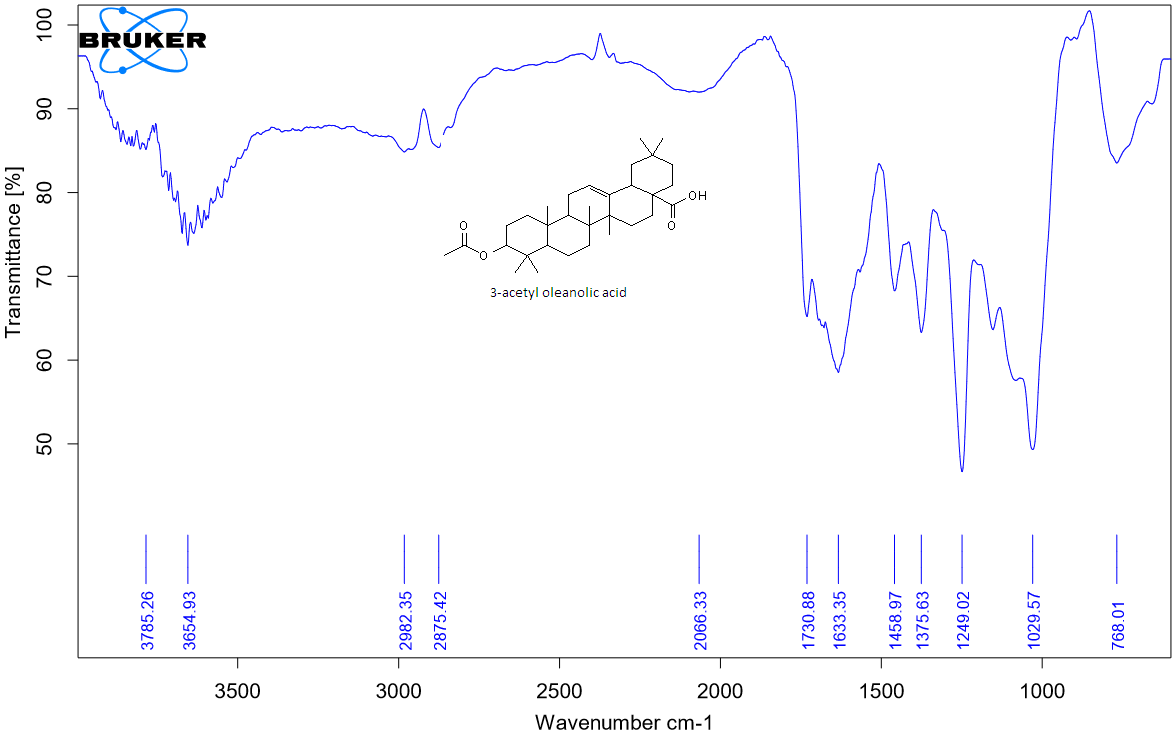
**

**Figure S20:** IR spectrum of 3-acetyl oleanolic acid

**S-4.5. 3-Phthaloyal oleanolic acid (Figure S21 – S24)**

Reactants: Oleanolic acid and phthalic anhydride

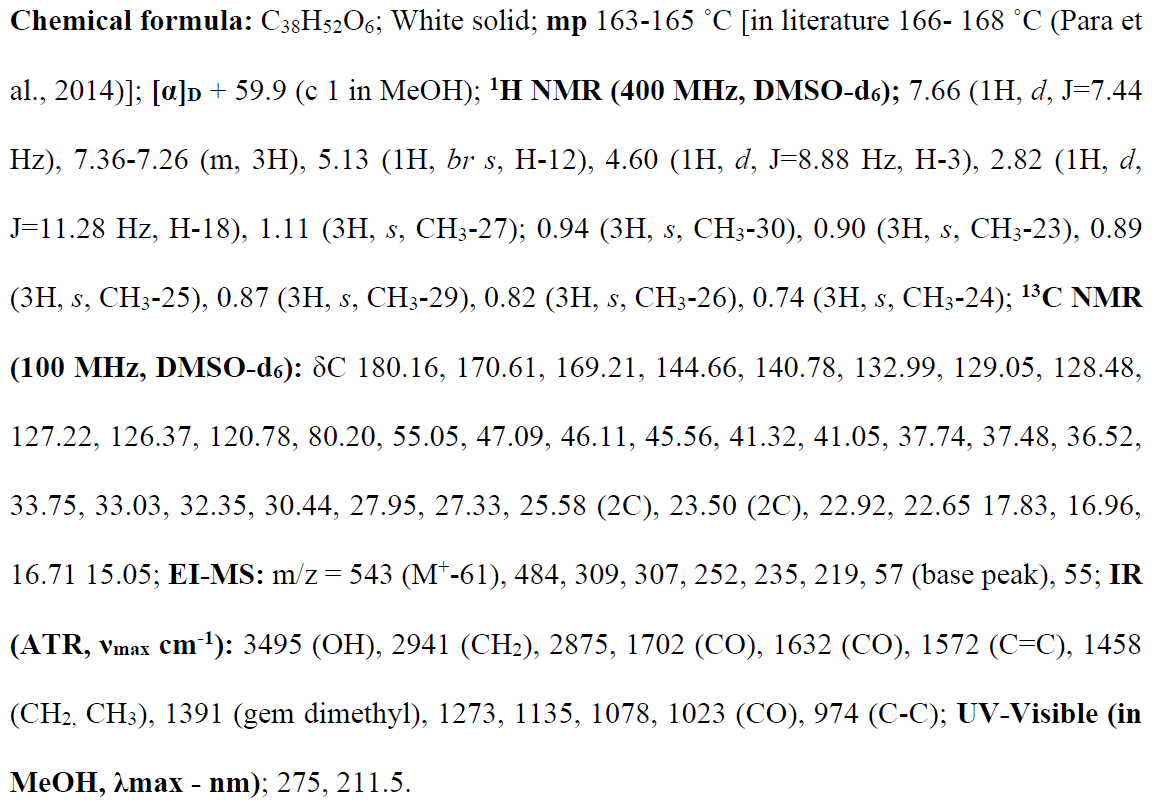


**
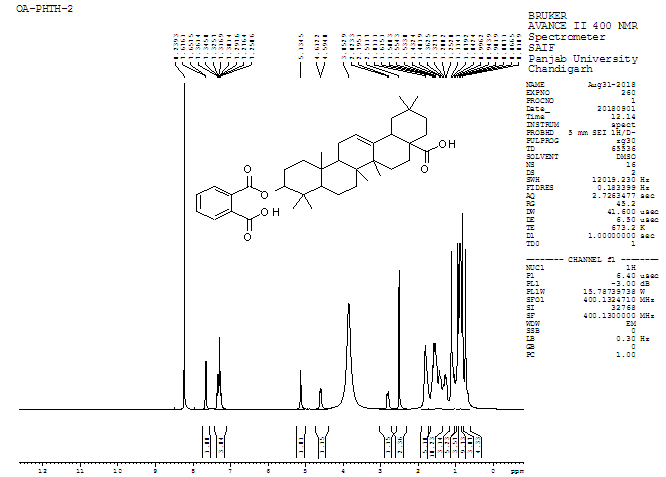
**

**Figure S21:** ^1^H NMR spectrum of 3-(phthalic acid)-oleanolic acid

**
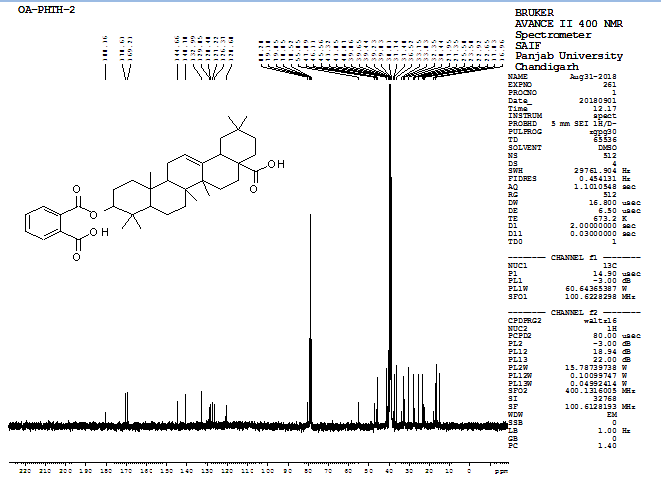
**

**Figure S22:** ^13^C NMR spectrum of 3-(phthalic acid)-oleanolic acid

**
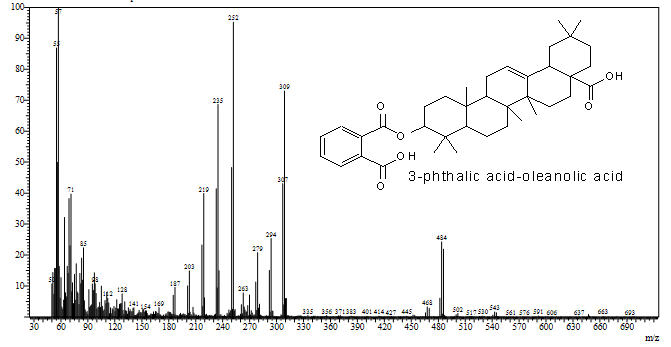
**

**Figure S23:** Mass spectrum of 3-(phthalic acid)-oleanolic acid

**
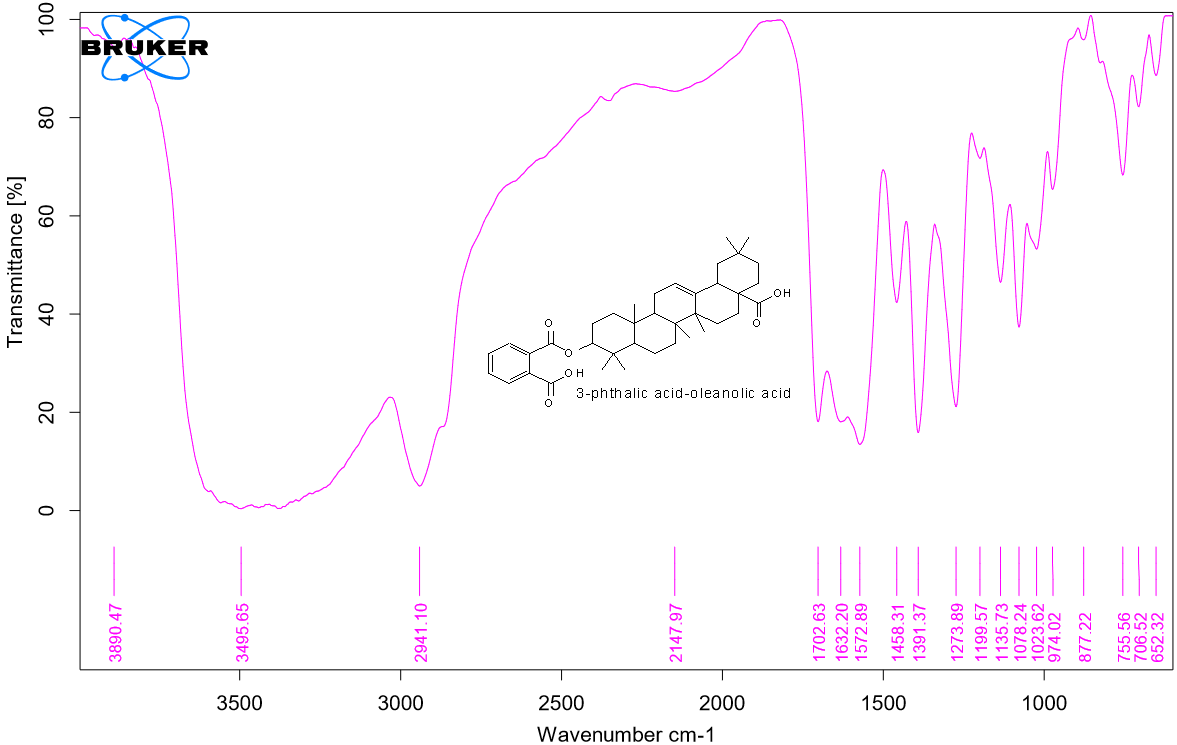
**

**Figure S24:** IR spectrum of 3-(phthalic acid)-oleanolic acid

**S-4.6. 3-Oxo oleanolic acid (OOA) (Figure S25 – S28)**

Reactants: Oleanolic acid and Jone’s reagent

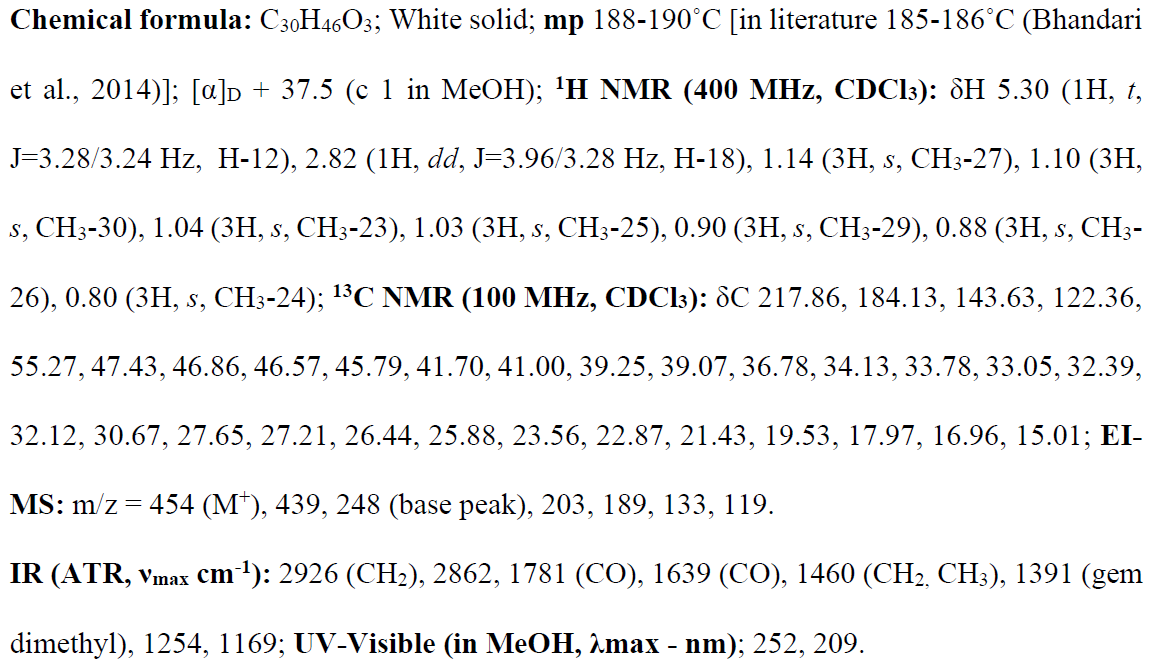


**
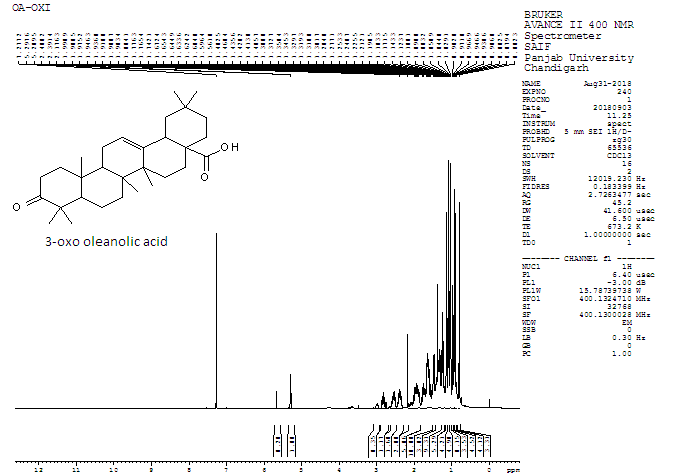
**

**Figure S25:** ^1^H NMR spectrum of 3-oxo oleanolic acid

**
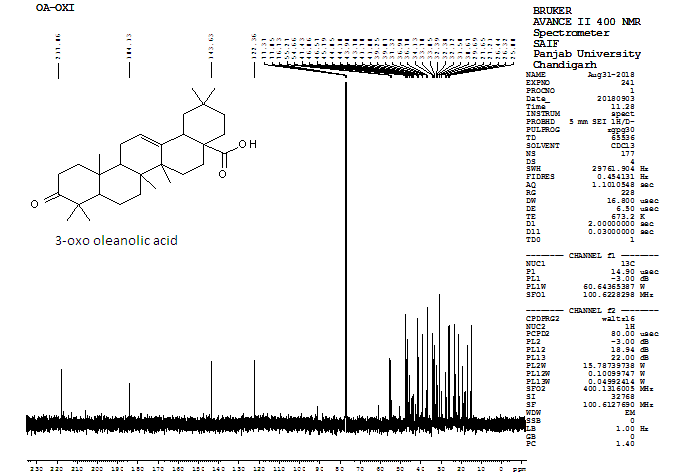
**

**Figure S26:** ^13^C NMR spectrum of 3-oxo oleanolic acid

**
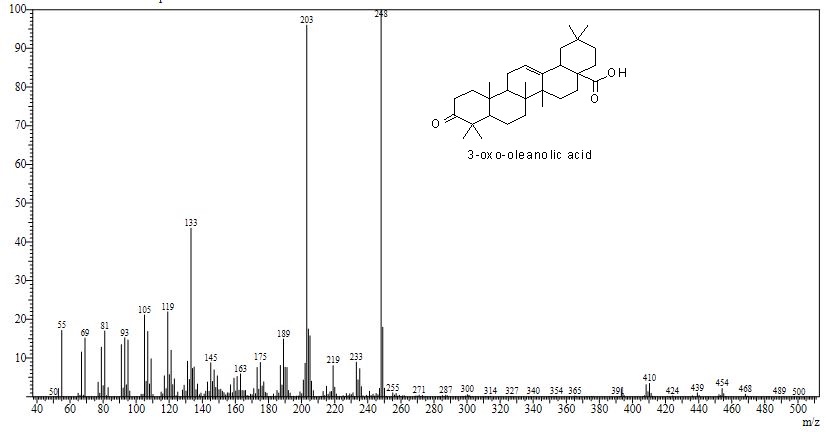
**

**Figure S27:** Mass spectrum of 3-oxo oleanolic acid

**
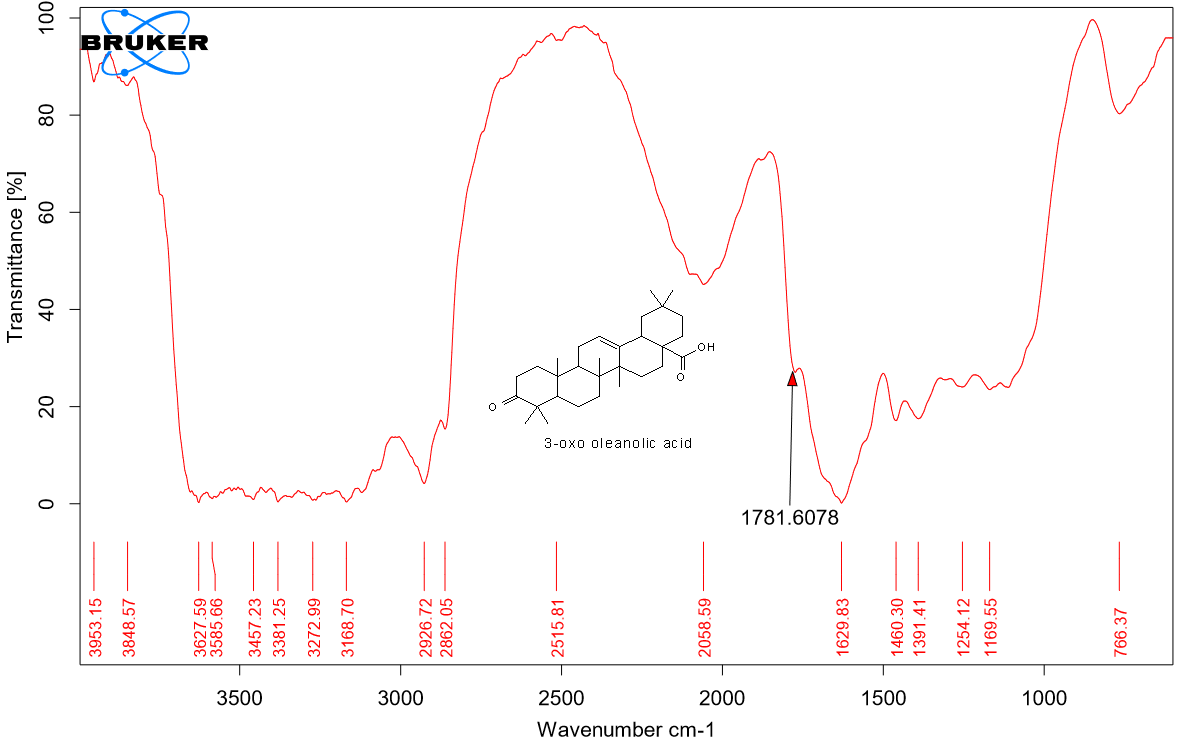
**

**Figure S28:** IR spectrum of 3-oxo oleanolic acid

**S-5. Result and Discussion**

**S-5.1. Isolation of pure compounds from CF**

**Oleanolic Acid**

In its ^1^H NMR**,** a triplet was observed at δ 5.27 ppm due to olefinic proton at C-12, doublet of doublet at δ 3.22 ppm due to proton at C-3, again doublet of doublet at δ 2.82 ppm for proton at C-18 and seven singlets at δ 1.13, 0.98*,* 0.93, 0.91, 0.90, 0.77 and 0.75 ppm due to seven methyl groups. Further, in its ^13^C NMR**,** peak at δ 183.52 ppm was observed correspond to carbonyl carbon (C-28), peak at δ 143.60 (C-13) and δ 122.63 ppm (C-12) were due to olefinic carbons. Further, peak due to carbon having hydroxyl group (C-3) was observed at δ 79.04 ppm and peak for C-5 was observed at δ 55.19 ppm. The IR spectral data (cm^-1^) of CF-2 showed peak at 3432 cm^-1^ due to OH group, at 1691 cm^-1^ due to CO group and at 1375 cm^-1^ due to gem dimethyl. Further, the DEPT 90 and 135 of crude CF-2 sample also revealed the presence of five CH, ten CH_2_ and seven CH_3_ group, which also supported the structure of oleanolic acid (Figure S8a and S8b in supplementary file). CF-3 also provides positive (+) Liebermann-Burchard test for triterpenoids, the compound could not be characterized as amount was very small (3.1 mg).

**S-5.2. Isolation of pure compounds from HF**

**Squalene**

In its ^1^H NMR, (Figure S9) multiplet for six olefinic protons was observed at δ 5.20-5.12 ppm and multiplet at δ 2.16-2.00 ppm was observed due to twenty methylene protons. Further, a broad singlet at δ 1.72 ppm was due to two terminal methyl protons, while broad singlet for six methyl protons was observed at δ 1.64 ppm. In its ^13^C NMR (Figure S10), twelve olefinic carbons appeared at δ 135.05-124.31 ppm. The peaks at δ 39.78-26.69 ppm were due to ten methylene carbons, while peaks corresponding to eight methyl carbons were appeared at δ 25.68-15.98 ppm. The IR spectral data of HF-2-1 (Figure S11) showed peaks (cm^-1^) at 2968, 2953 and 2853 cm^-1^ due to C-H stretching of CH_2_ and CH_3_, peak at 1666 cm^-1^ correspond to olefinic stretching (C=C) and peaks at 1443, 1216 and 1152 cm^-1^ were due to C-H banding of CH_2,_ CH_3_ and CH groups. On the basis of ^1^H NMR, ^13^C NMR, mass and IR data HF-2-1 was identified as squalene (Figure S9 – S12 in the supplementary data file).

The GC-MS analysis of HF-3-1 showed one major peak (92%) correspond to abieta-9(11), 8(14), 12-trien-12-ol (Figure 4.13). The isolated amount of HF-3-1 is very small (1.9 mg). The diterpenoids with abietane structure were also reported previously in various extracts obtained from different *Nepeta* species (Formisano et al., 2011; Fraga et al., 2017; Suntar et al., 2017; Sharma et al., 2021a). HF-5 was the mixture of a number of components, which were difficult to separate due to small amount (8.3 mg). The GC-MS analysis of HF-5 showed the presence of menthyl acetate (44.49%), hexadecenoic acid (20.51%) and octadec-9-enoic acid (13.17%) as the key components. HF-5 was the mixture of a number of components, which were difficult to separate due to small amount (8.3 mg).

In its ^1^H NMR**,** multiplet for six olefinic protons was observed at δ 5.39-5.29 ppm, singlet due ester methyl group was appeared at δ 3.22 ppm. A triplet due to two methylene groups present between two C-C double bond appeared at δ 2.80 ppm, while triplet due to two protons (C**H_2_**COOR) was observed at δ 2.29 ppm. Further, multiple corresponding to two proton (CH_3_C**H_2_**CH=CH) appeared at δ 2.09-2.02 ppm, while a multiplet corresponding to four methylene groups was observed at δ 1.36-1.31 ppm. A triplet at δ 0.97 ppm was due to terminal methyl group. In its ^13^C NMR**,** peak at δ 174.16 ppm was due to carbonyl carbon and six peaks at δ 131.87 - 127.70 ppm were due to six olefinic carbons. The peak due to carbon of methyl group attached to oxygen atom appeared at δ 51.33 ppm, while peak at δ 14.21 ppm corresponds to carbon atom of terminal methyl group. The rest of the peaks at δ 34.03-20.51 ppm were due to various methylene (CH_2_) carbon atoms. The IR spectral data of HF-7-2, showed peak at 3007 cm^-1^ due to olefinic C-H stretching. The peaks at 2927 and 2858 cm^-1^ were correspond to CH_2_ groups and peak due ester group appeared at 1733 cm^-1^. The GC-MS analysis of HF-11 showed one major peak with 87.1% abundance (Figure S31). HF-11 was not characterized due to the small amount of material isolated. The GC-MS analysis revealed that the compound would be a diterpene derivative (library hit).

**
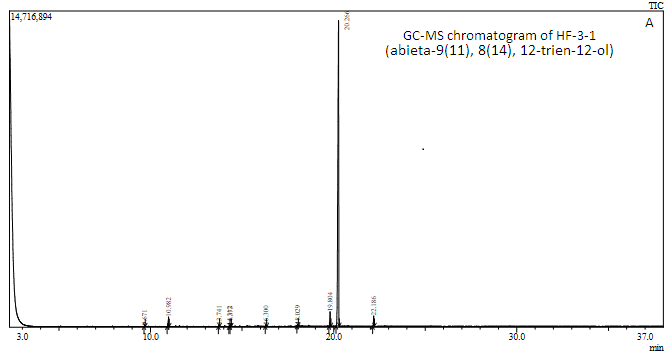
**

**
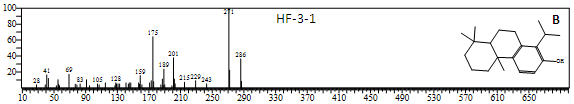
**

**Figure S29. A)** GC-MS chromatogram of HF-3-1 **B)** Mass spectrum of HF-3-1 (Similarity Index 84%)

**
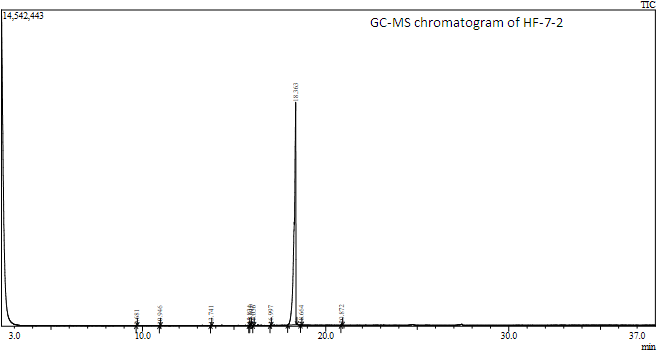
**

**Figure S30.** GC-MS chromatogram of HF-7-2


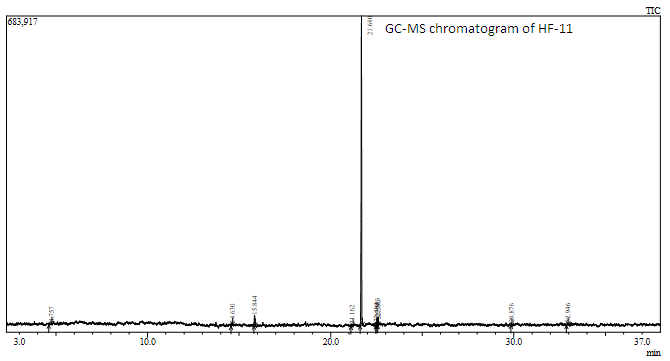


**Figure S31.** GC-MS chromatogram of HF-11

**S-5.3. Derivatization of isolated bioactive compounds and evaluation of antioxidant potential of synthesized derivatives**

**3-acetyl-oleanolic acid**

In its ^1^H NMR (Figure S17), a triplet due to one proton at C-3 appeared at down field δ 4.49 ppm [as compared to same peak of C-3 proton (δ 3.22 ppm) of oleanolic acid], Another singlet at δ 2.05 ppm due to three proton of H_3_CCOO was observed, which was absent in oleanolic acid. Similarly, in ^13^C NMR of the product (Figure S18), two added peaks at δ 171.10 ppm due to C=O group of acetyl moiety and at δ 21.20 ppm due to methyl carbon of acetyl group was observed compared to the ^13^C peaks of oleanolic acid. The rest of the ^1^H NMR and ^13^C NMR data was similar to the oleanolic acid. In its IR (cm^-1^) spectrum, it showed bands/peaks at 2982-2875 cm^-1^ due to aliphatic CH stretching, 1730 cm^-1^ due to C=O of ester linkage, 1633 cm^-1^ due to C=O of carboxylic group, 1375 cm^-1^ due to gem dimethyl group (Figure S20).

**3-Phthaloyl oleanolic acid**

The ^1^H NMR of product showed a doublet due to one proton of C-3 at δ 4.60 ppm as compared to the same peak of oleanolic acid, which appeared at δ 3.22 ppm. Further, new peaks due to aromatic protons appeared at δ 7.66 (1H, *d*, J=7.44 Hz) and δ 7.36-7.26 ppm (m, 3H), which were absent in case of oleanolic acid. Further, in the ^13^C NMR**,** two added peaks due to carbonyl groups appeared at δ 170.61 and 169.21 ppm, while six new peaks due to aromatic carbons appeared at δ 140.78, 132.99, 129.05, 128.48, 127.22 and 126.37 ppm. IR spectrum of product showed peaks at 3495 cm^-1^ due to hydroxyl group, at 2941 cm^-1^ and 2875 cm^-1^ correspond to CH_2_ groups. The peaks at 1702 and 1632 cm^-1^ were due the carbonyl group, while peak due to gem dimethyl group appeared at 1391 cm^-1^.

**3-oxo-oleanolic acid**

In its ^1^H NMR (Figure S25)**,** a peak due to one proton at C-3 was not observed in the spectrum, which otherwise appeared at δ 3.22 ppm in the ^1^H NMR of oleanolic acid. The rest of the spectrum of 3-oxo-oleanolic acid was almost similar to that of oleanolic acid. Further, in its ^13^C NMR (Figure S26)**,** a peak due to new carbonyl group appeared at δ 217.8 ppm, while a peak for the carbonyl group of acid group was observed at δ 184.13 ppm. The peak at δ 79.04 ppm due to C-3 (observed in case of oleanolic acid) was observed at δ 217.8 ppm due to C=O at C-3. 3-Oxo-oleanolic acid showed an IR peak (Figure S28) at 2926 and 2862 cm^-1^ corresponding to CH_2_ groups, at 1781 and 1639 cm^-1^ due to carbonyl group. The band due to gem dimethyl group appeared at 1391 cm^-1^


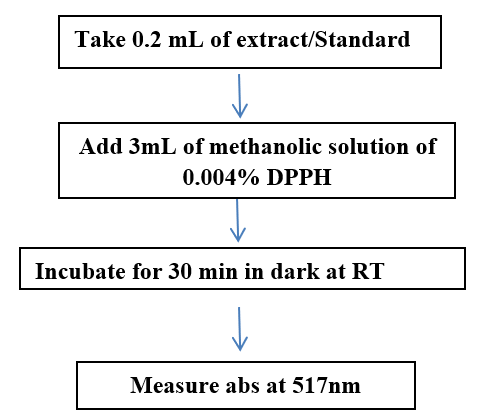


**Figure S32.** The detail of method followed in DPPH assay


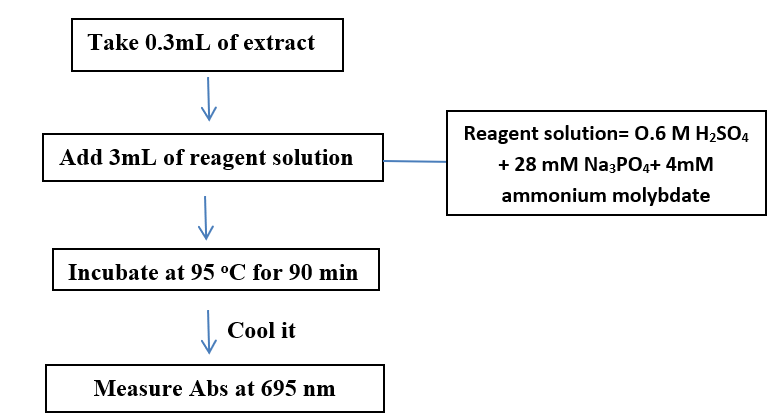


**Figure S33.** The detail of method followed in TAC assay

**S-6. In-silico Studies**

**Table S1:** Properties of selected cavities of target proteins

| Target protein | Position | | | Volume  (Å3) | Surface  (Å2) |
| --- | --- | --- | --- | --- | --- |
|  | X | Y | Z |  |  |
| arabinosyl transferase (PDB ID 3PTY) | 95.7802 | 6.44418 | 6.51289 | 92.672 | 337.92 |
| Enoyl acyl reductase (PDB ID 1C14) | -10.984 | 41.5965 | 156.624 | 340.992 | 775.68 |
| FtsA (PDB ID 3WQU) | 23.1079 | 5.4184 | 15.8486 | 416.256 | 1492.48 |
| reverse transcriptase (PDB ID 3V4I) | -12.5598 | -37.2572 | 38.25 | 4395.52 | 10796 |
| hemagglutinin (PDB ID 1RUZ) | 44.7063 | 82.2521 | 63.972 | 4399.1 | 8056.32 |
| COVID-19 main protease (PDB ID 6LU7) | -10.8047 | 15.5231 | 68.3383 | 118.784 | 391.68 |

**
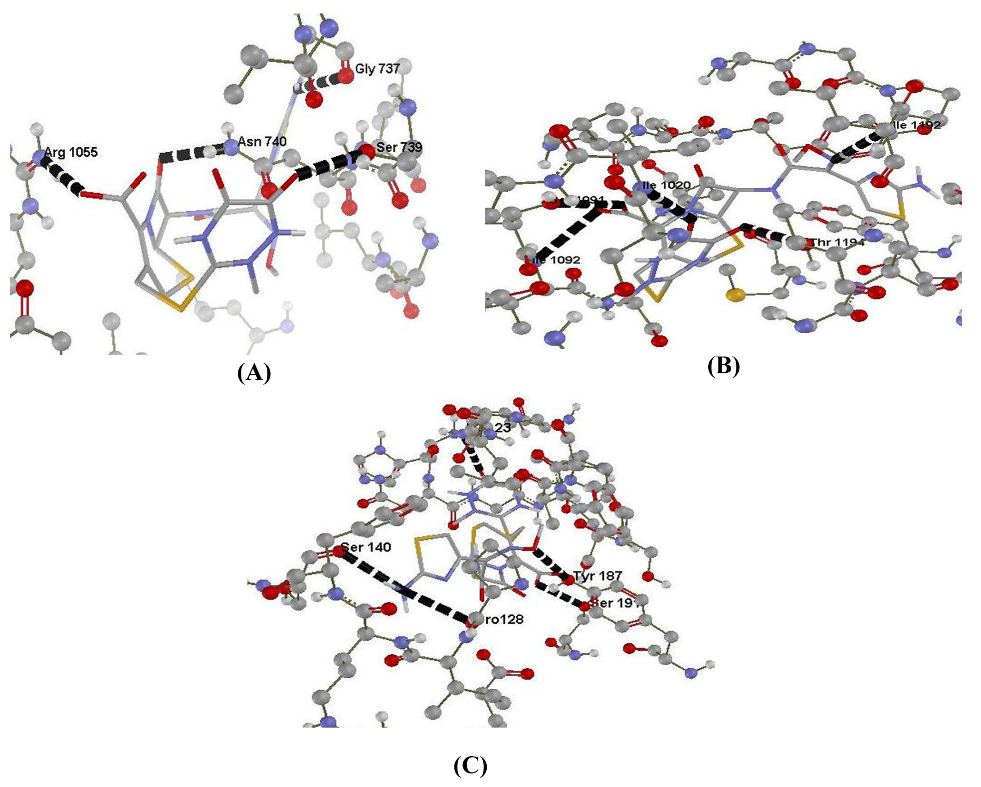
**

**Figure S34.** H-Bond pattern of dug Ceftriaxone with arabinosyl transferase (*a)*, Enoyl acyl reductase (*b) and* FtsA (*c*) with interacting residues


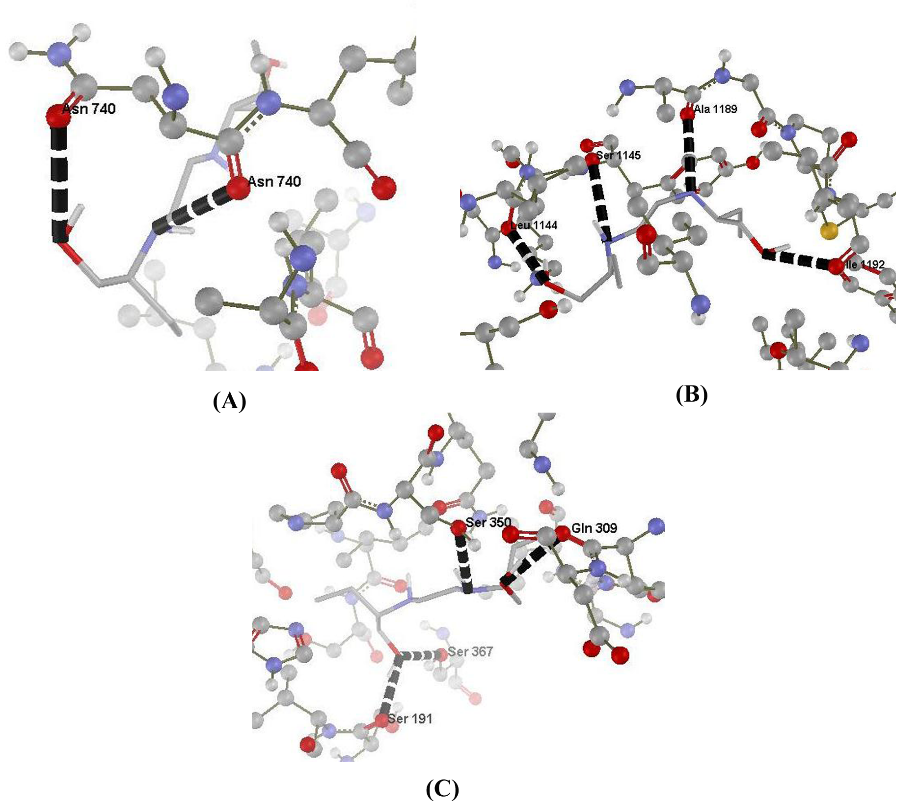


**Figure S35.** H-Bond pattern of drug Ethambutol with arabinosyl transferase (*a)*, Enoyl acyl reductase (*b) and* FtsA (*c*) with interacting residues


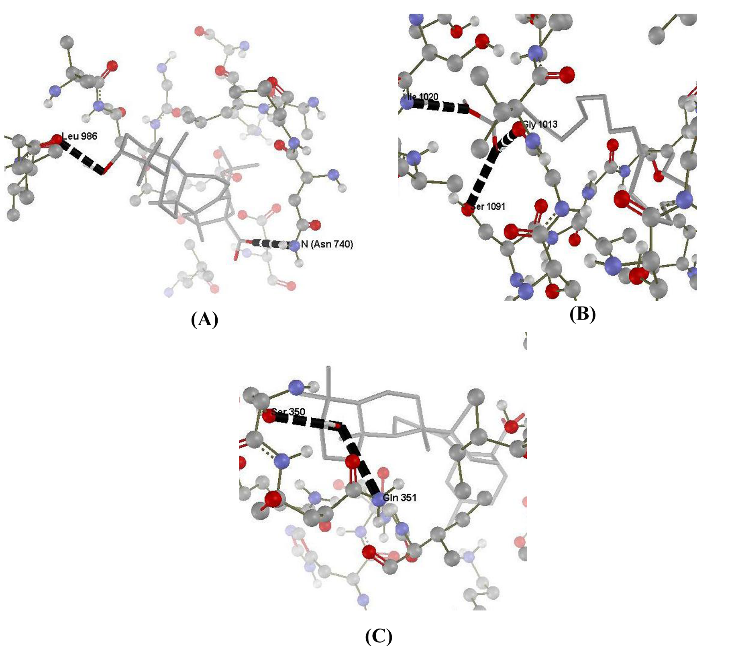


**Figure S36.** H-Bond pattern of Oleanolic acid with arabinosyl transferase (*a)*, Enoyl acyl reductase (*b) and* FtsA (*c*) with interacting residues


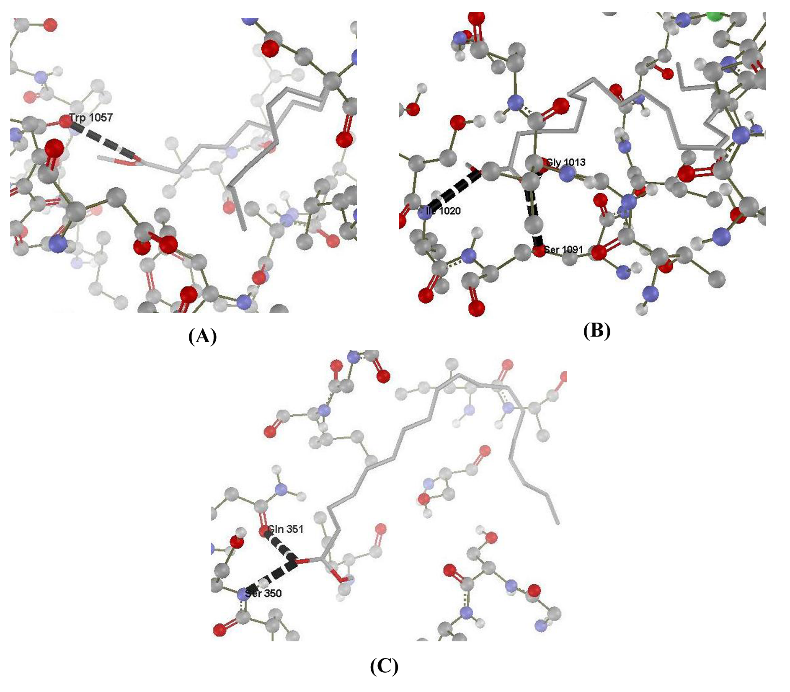


**Figure S37.** H-Bond pattern of Linolenic acid methyl ester with arabinosyl transferase (*a)*, Enoyl acyl reductase (*b) and* FtsA (*c*) with interacting residues


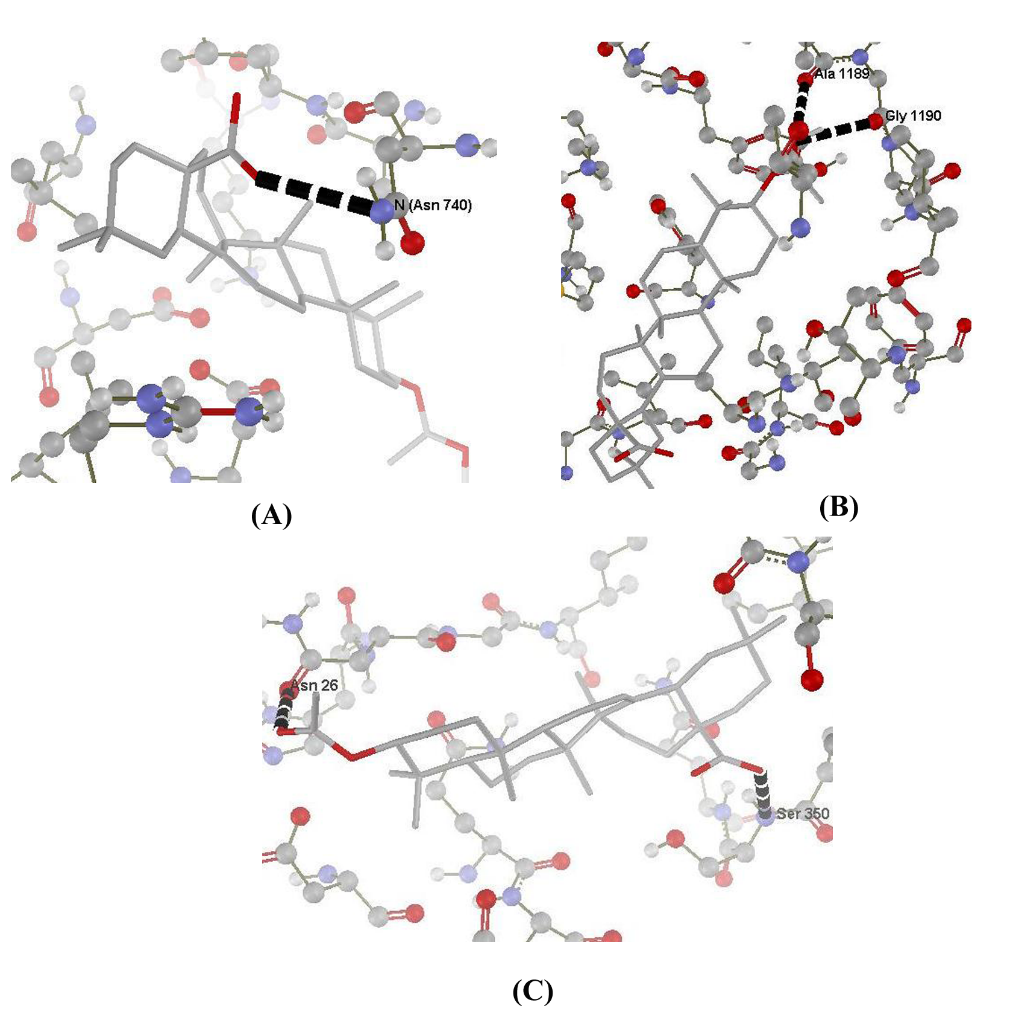


**Figure S38.** H-Bond pattern of 3-Acetyl oleanolic acid with arabinosyl transferase (*a)*, Enoyl acyl reductase (*b) and* FtsA (*c*) with interacting residues


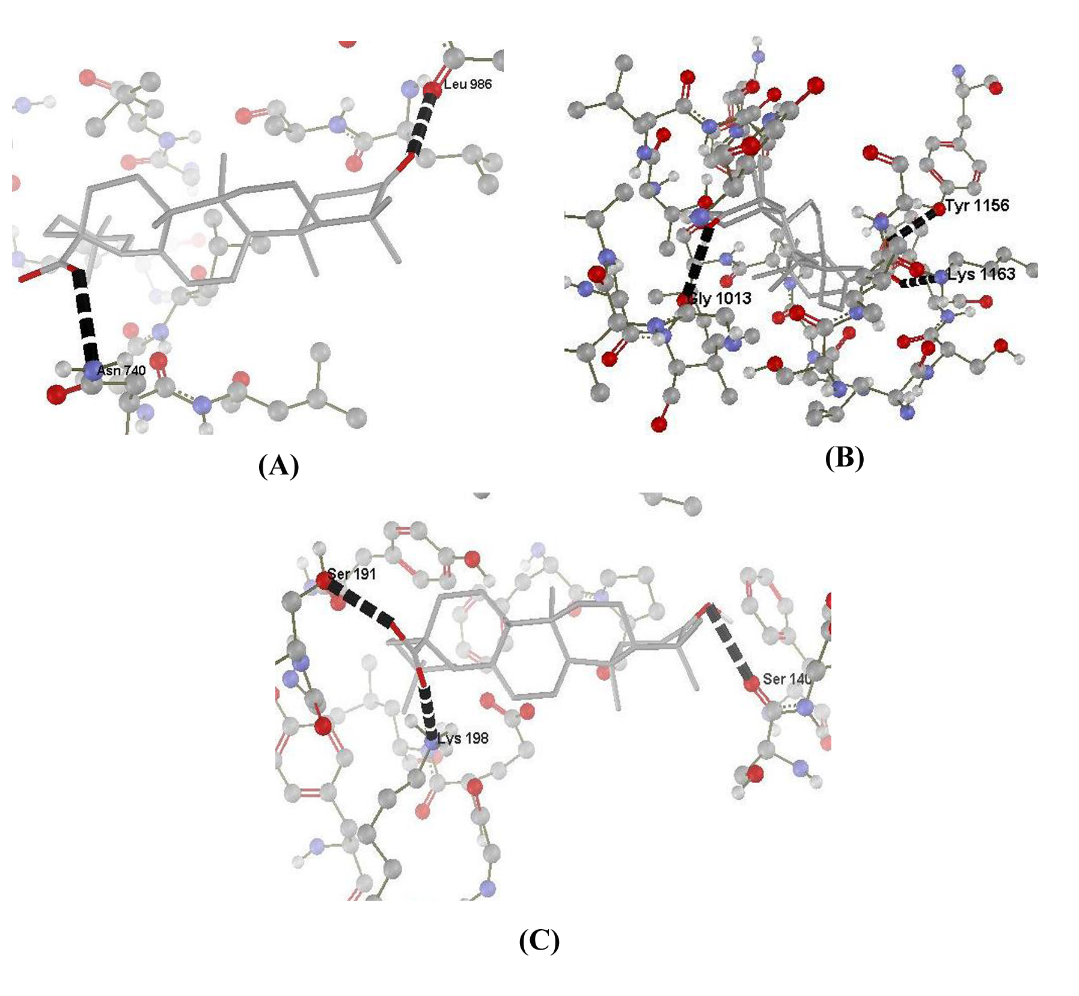


**Figure S39.** H-Bond pattern of 3-Oxo oleanolic acid with arabinosyl transferase (*a)*, Enoyl acyl reductase (*b) and* FtsA (*c*) with interacting residues


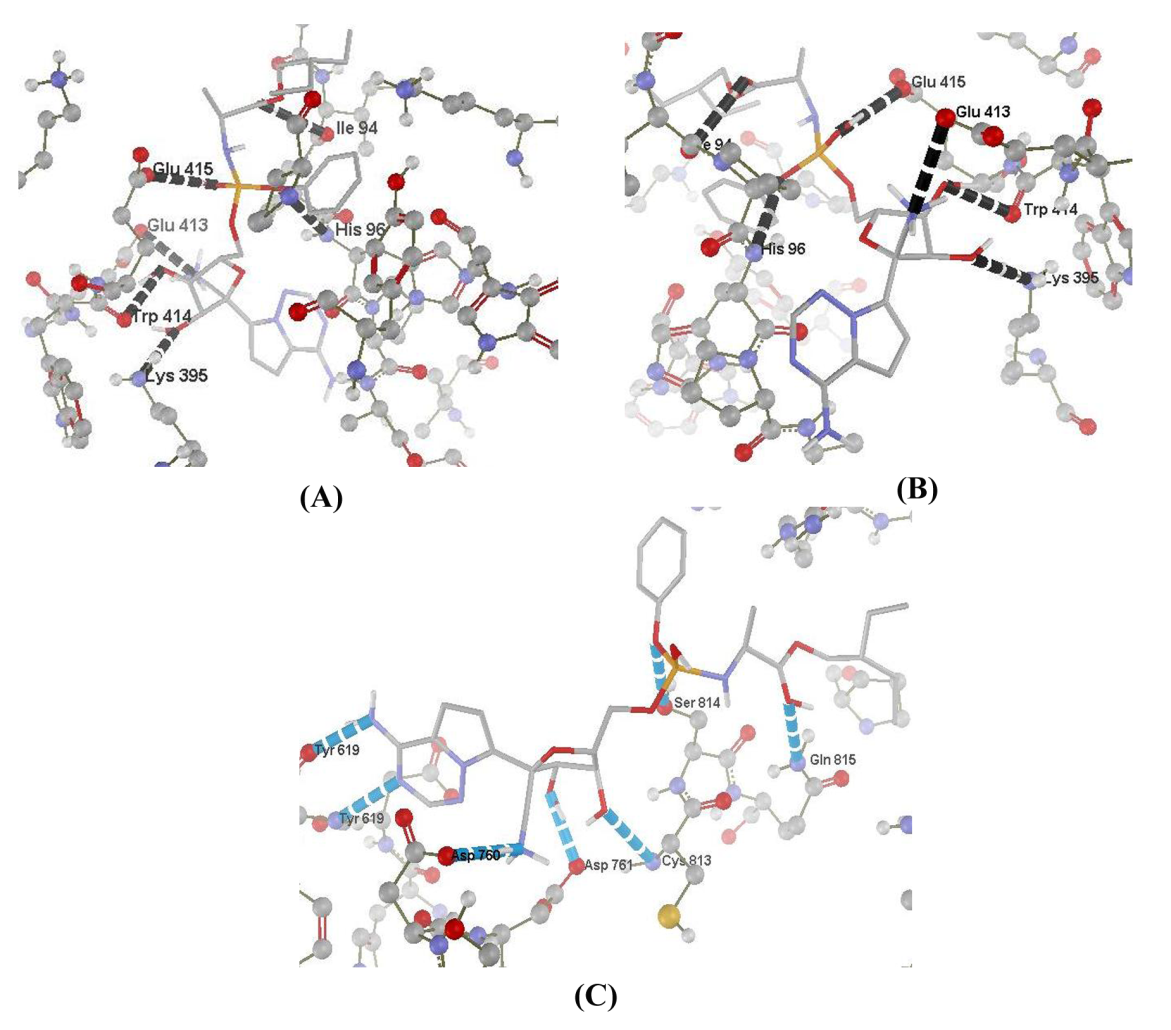


**Figure S40.** H-Bond pattern of drug remdesivir with hemagglutinin (a),reverse transcriptase (b) and RNA dependant RNA polymerase (c) with interacting residues


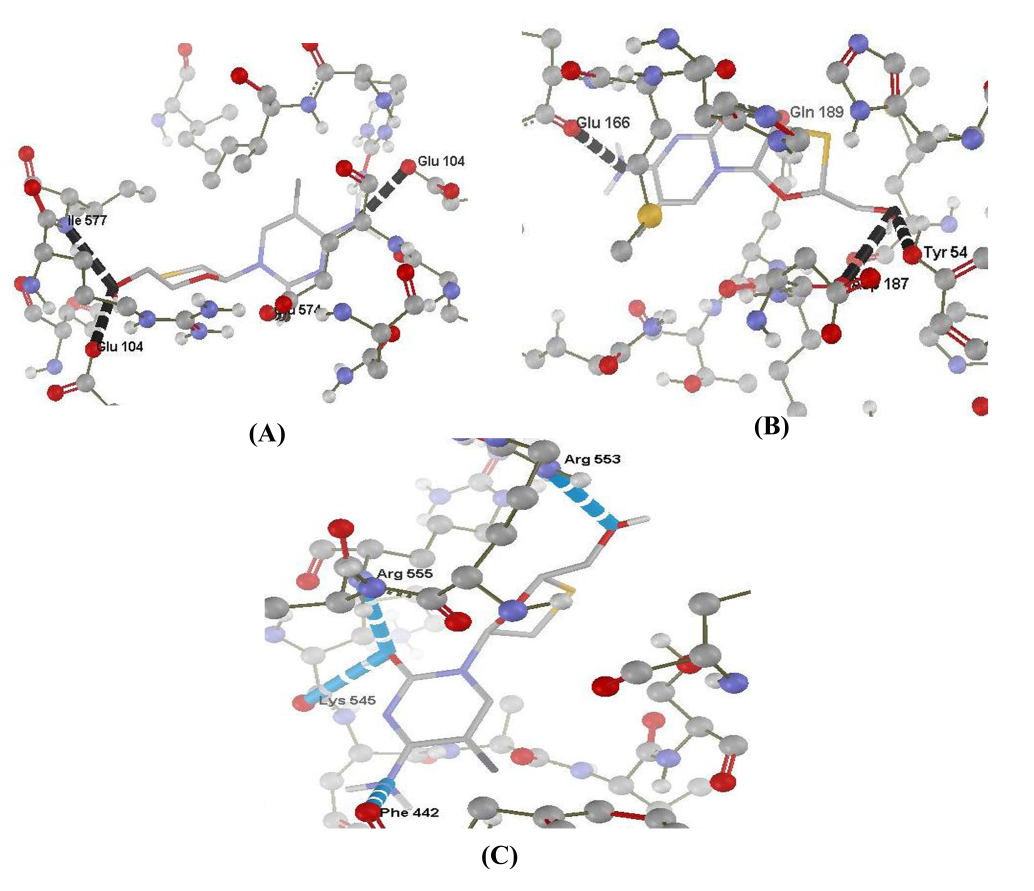


**Figure S41.** H-Bond pattern of drug Emtricitabine with hemagglutinin (a), reverse transcriptase (b) and RNA dependant RNA polymerase (c) with interacting residues


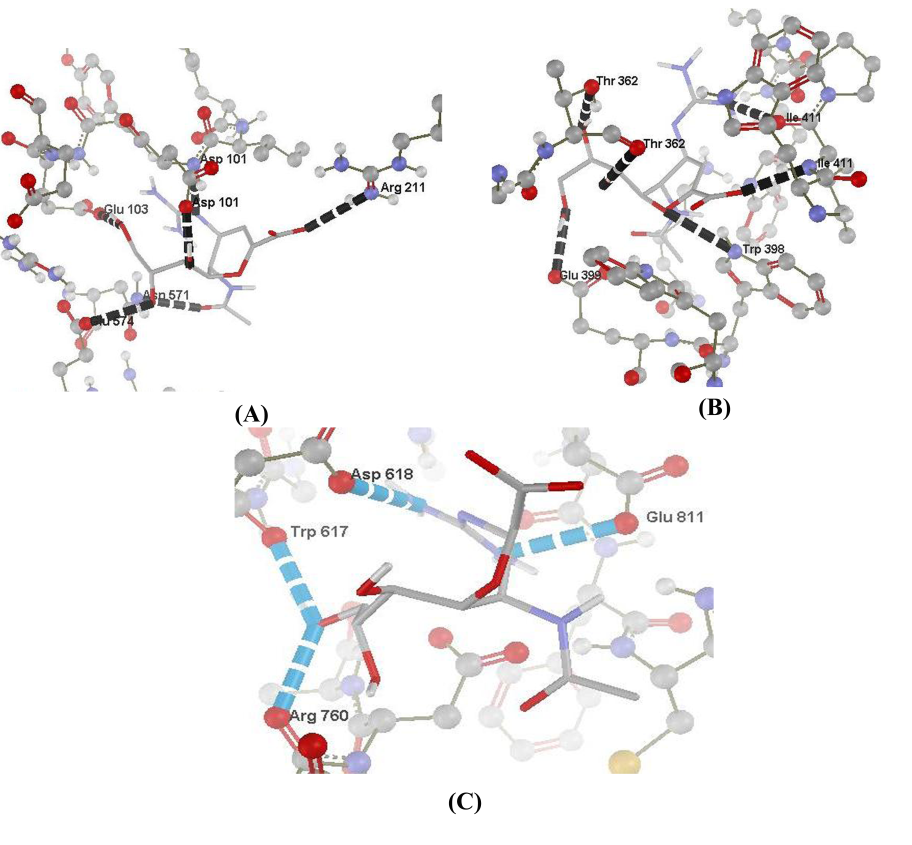


**Figure S42.** H-Bond pattern of drug Zanamivir with hemagglutinin (a), reverse transcriptase (b) and RNA dependant RNA polymerase (c) with interacting residues.

**
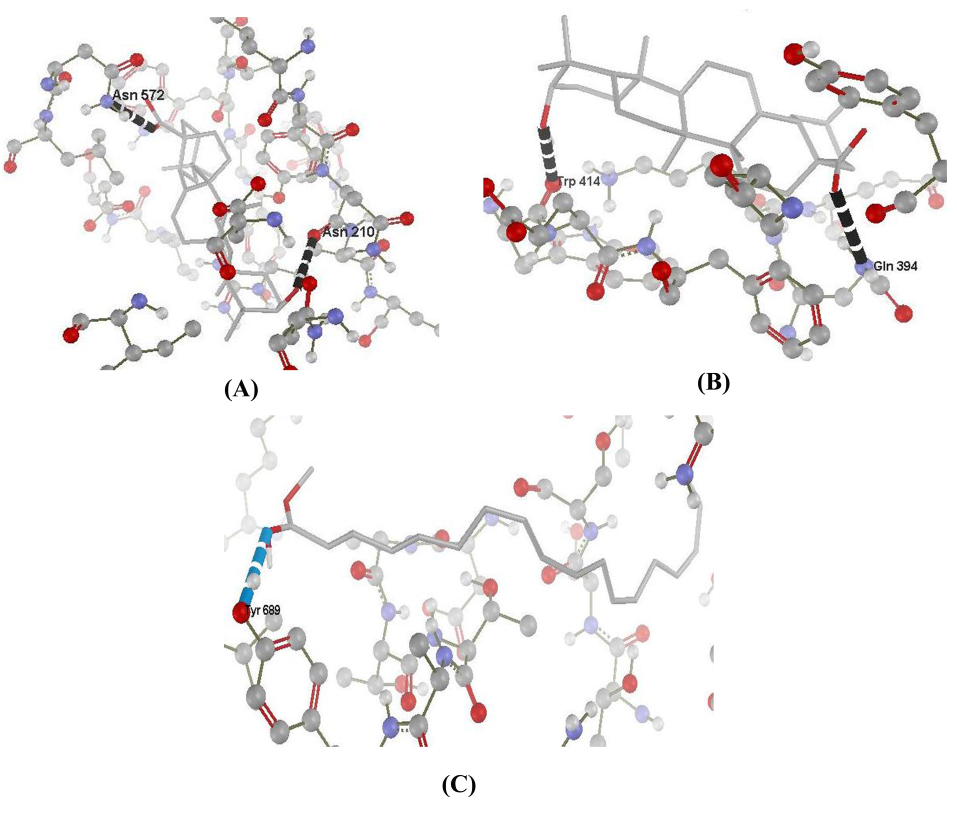
**

**Figure S43.** H-Bond pattern of Oleanolic acid with hemagglutinin (a), reverse transcriptase (b) and RNA dependant RNA polymerase (c) with interacting residues


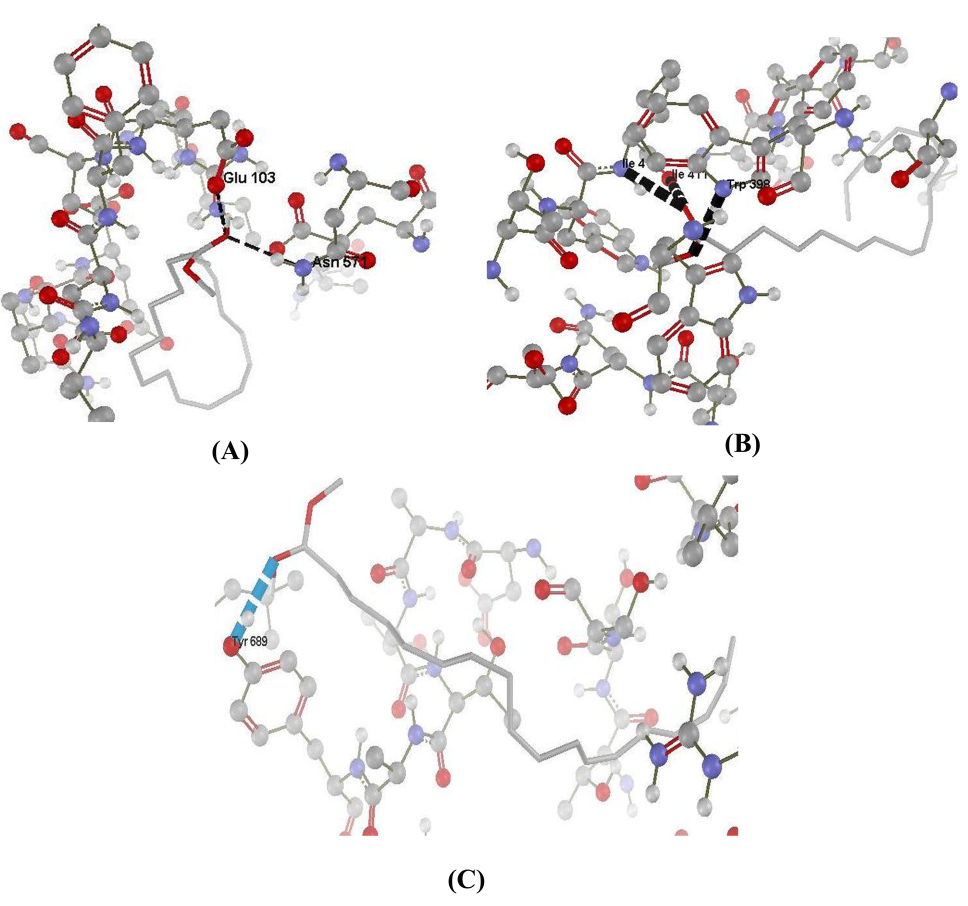


**Figure S44** H-Bond pattern of Linolenic acid methyl ester with hemagglutinin (a), reverse transcriptase (b) and RNA dependant RNA polymerase (c) with interacting residues


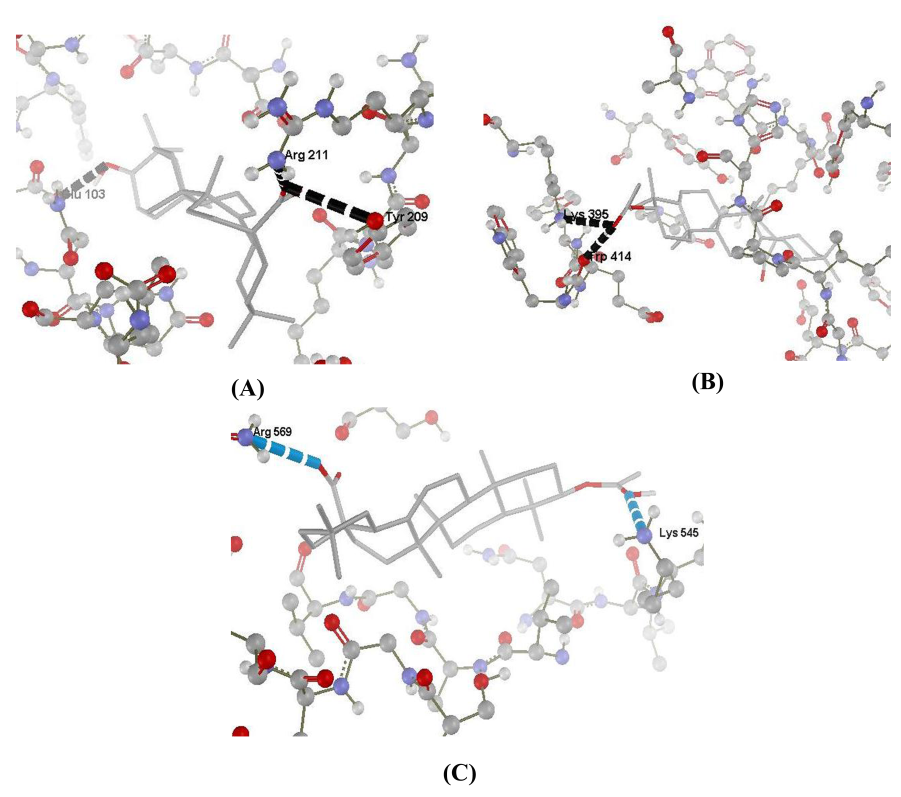


**Figure S45.** H-Bond pattern of 3-Acetyl oleanolic acid with hemagglutinin (a), reverse transcriptase (b) and RNA dependant RNA polymerase (c) with interacting residues


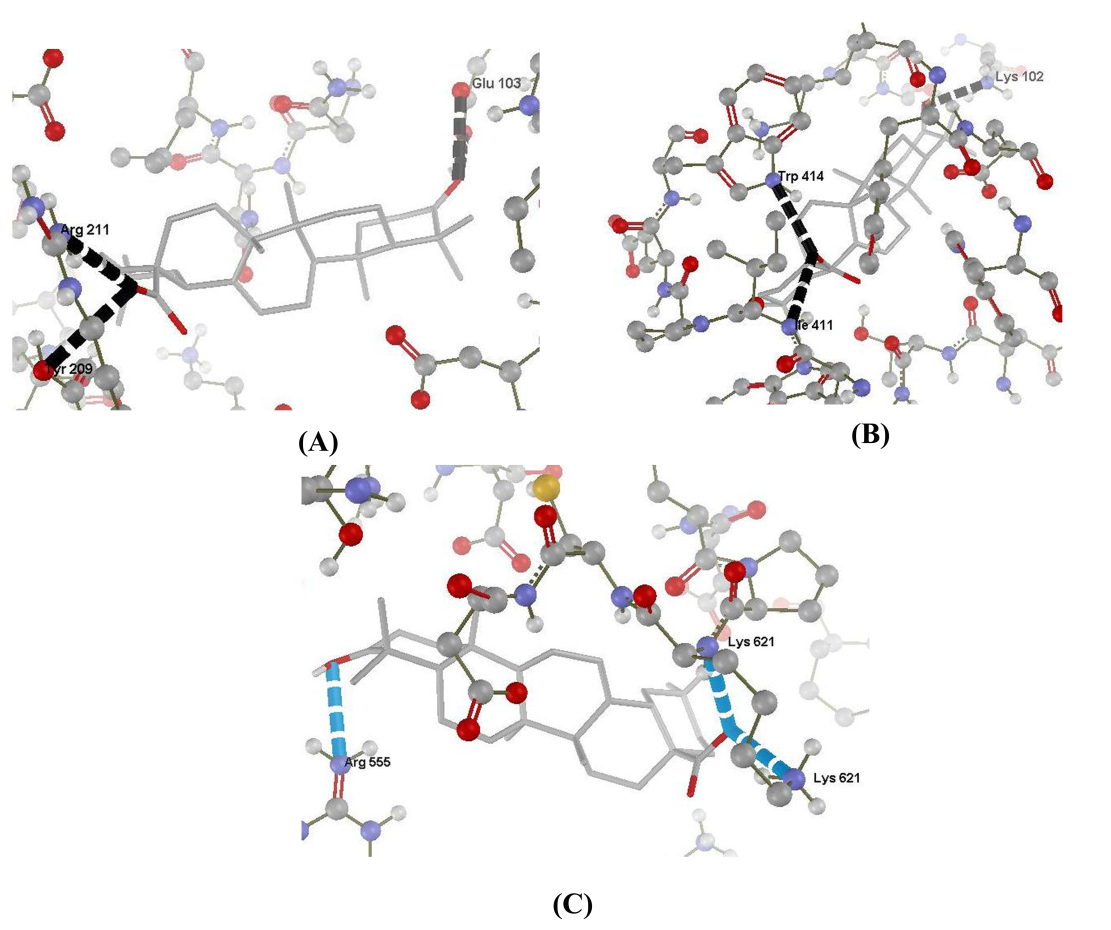


**Figure S46.** H-Bond pattern of 3-Oxo oleanolic acid methyl ester with hemagglutinin (a), reverse transcriptase (b) and RNA dependant RNA polymerase (c) with interacting residues

| **Table S2.** Hydrogen bonding pattern at the binding sites of the three antibacterial targets | | | |
| --- | --- | --- | --- |
| Compound | H-bond with arabinosyltransferase (*Mycobactrium tuberculosis*) | H-bond with Enoyl acyl reductase (*E-coli)* | H-bond with FtsA (*Staphylococcus aureus*), |
| *Ceftriaxone | N(Arg 1055)-H-O  N(Asn 740)-H-O  O(Gly 737)-H-N  O(Ser 739)-H-O | O( Ser 1091)-H-O  N( IIe 10920-H-O  N( IIe 1020)-H-O  N( IIe 1192)-H-N  O( Thr 1194)-H-O | O( Pro128)-H-N  O( Ser 140)-H-N  O( Tyr 187)-H-O  O( Ser 191)-H-O  N( Lys 23)-H-O |
| Squalene | Nil | Nil | Nil |
| 3-Phthaloyl oleanolic acid | N( Asn 740)-H-O | N( Gln 1040)-H-O  N( Gly 1093)-H-O  O( Tyr 1156)-H-O  N( Lys 11630-H-O | N( IIe 28)-H-O  O( Tyr 187)-H-O  O( Ser 191)-H-O  N(Gln 351)-H-O  O(Ser 368)-H-O |
| Linolenic acid methyl ester | O(Trp 1057)-H-O | N(IIe 1020)-H-O  O(Gly 1013)-H-O  O(Ser 1091)-H-O | N(Ser 350)-H-O  O(Gln 351)-H-O |
| 3-Oxo oleanolic acid | N( Asn 740)-H-O;  O(Leu 986)-H-O | O( Gly 1013)-H-O  O( Tyr 1156)-H-O  N( Lys 1163)-H-O | N( Lys 198)-H-O  O( Ser 191)-H-O  O( Ser 140)-H-O |
| 3-Acetyl oleanolic acid | N(N (Asn 740)-H-O | O(Gly 1190)-H-O  O(Ala 1189)-H-O | O(Asn 26)-H-O  N(Ser 350)-H-O |
| Oleanolic acid | O( Leu 986)-H-O  N (Asn 740)-H-O | N(IIe 1020)-H-O  O(Ser 1091)-H-O  O(Gly 1013)-H-O | O(Ser 350)-H-O  N(Gln 351)-H-O |
| *Ethambutol | O( Asn 740)-H-O  O( Asn 740)-H-N | O( Ala 1189)-H-N  O( IIe 1192)-H-O  O( Ser 1145)-H-N  O( Leu 1144)-H-O | O( Gln 309)-H-O  O( Ser 350)-H-N  O( Ser 367)-H-O  O( Ser 191)-H-O |
| *antibacterial drug compound | | | |

| **Table S3.** Hydrogen bonding pattern at the binding sites of the three antiviral targets | | | |
| --- | --- | --- | --- |
| Compound | H-bond with hemagglutinin (H1N1) | H-bond with reverse transcriptase (HIV) | H-bond with RNA dependant RNA polymerase |
| * remdesivir | O(Glu 216)-H-O  N(Asn 210)-H-O  O(Arg 212)-H-N  N(His 184-H-O  O(Asp 98)-H-O  N(Asn 231)-H-O  N(Thr 214)-H-O | O( Glu 415)-H-O  O( IIe 94)-H-O  N( His 96)-H-O  O( Glu 413)-H-N  O( Trp 414)-H-O  N( Lys 395)-H-O | O(Tyr 619)-H-N  N(Tyr 619)-H-N  O(Ser 814)-H-O  N(Gln 815)-H-O  O(Asp 760)-H-N  N(Cys 813)-H-O  O(Asp 761)-H-O |
| Squalene | Nil | Nil | Nil |
| 3-Phthaloyl oleanolic acid | N( Arg 211)-H-O  O( Tyr 213)-H-O  N( Asn 210)-H-O  N( Asn 231)-H-O | N( His 96)-H-O  O( His 96)-H-O  N( His 96)-H-O  N( Gly 93)-H-O | N( Arg 553)-H-O  O( Ser 549)-H-O  N( Asp 623)-H-O  S( Cys 622)-H-O  O( Ser 759)-H-O  N( Asn 691)-H-O |
| Linolenic acid methyl ester | O(Glu 103)-H-O  N(Asn 571)-H-O | N(Trp 398)-H-O  O(IIe 411)-H-O  N(IIe 411)-H-O | O( Tyr 689)-H-O |
| 3-Oxo oleanolic acid | N( Arg 211)-H-O  O( Tyr 209)-H-O  O( Glu 103)-H-O | N( Lys 102)-H-O  N( IIe 411)-H-O  N( Trp 414)-H-O | N ( Arg 555)-H-O  N( Lys 621)-H-O  N( Lys 621)-H-O |
| 3-Acetyl oleanolic acid | N(Arg 211)-H-O  O(Tyr 209)-H-O  N(Glu 103)-H-O | N(Lys 395)-H-O  O(Trp 414)-H-O | N( Arg 569)-H-O  N( Lys 545)-H-O |
| Oleanolic acid | O(Asn 210)-H-O  N(Asn 572)-H-IO | N(Gln 394)-H-O  O(Trp 414)-H-O | O ( Tyr 689)-H-O |
| *Emtricitabine | O( Glu 104)-H-N  O( Glu 574)-H-O  N( IIe 577)-H-O  O( Glu 104)-H-O | O( Trp 414)-H-O  O( His 361)-H-N  N( Trp 398)-H-O  N( Trp 402)-H-N | O( Lys 545)-H-O  N(Arg 555)-H-O  O( Phe 442)-H-O  N( Arg 553)-H-O  O( Phe 442)-H-O |
| *Zanamivir | N( Arg 211)-H-O  O( Asp 101)-H-O  O( Glu 574)-H-O  N( Asn 571)-H-O  N( Asp 101)-H-N  O( Glu 103)-H-O | O( Thr 362)-H-O  O( IIe 411)-H-O  O( Thr 362)-H-O  N( Trp 398)-H-O  O( Glu 399)-H-O  N( IIe 411)-H-O | O ( Arg 760)-H-O  O(Trp 617)-H-O  O( Asp 618)-H-O  O( Glu 811)-H-O |

**Compliance with ethical standards**

**Conﬂict of interest -** The authors have no conflicts of interest.

**Acknowledgements**

The authors gratefully acknowledge the financial support provided by the Department of Science and Technology, Govt. of India, New Delhi (INSPIRE Code IF120715). We also acknowledge Prof. M.I.S. Saggoo of Punjabi University, Patiala (Punjab), India for identifying the plant material.

**References**

Ribeiro, N.C., Demuner, A.J., Santos, M.H.D., Maltha, C.R.A., de Alvarenga, E.A., Komarnytsky, S. 2018. Metals complexes formed with oleanolic acid. Int J Org Chem. 8: 160-169.

Rowe, E.J., Orr, J.E., Uhl, A.H., Parks, L.M. 1949. Isolation of oleanolic acid and ursolic acid from Thymas vulgaris. J Am Pharma Assoc. 38: 122-124.

Bhandari, P., Patel, N.K., Gangwal, R.P., Sangamwar, A.T., Bhutani, K.K. 2014. Oleanolic acid analogs as NO, TNF-a and IL-1b inhibitors: synthesis, biological evaluation and docking studies. Bioorg Med Chem Lett. 24: 4114–4119.

Rali, S., Oyedeji, O.O., Aremu, O.O., Oyedeji, A.O., Nkeh-Chungag, B.N. 2016. Semisynthesis of derivatives of oleanolic acid from *Syzygium aromaticum* and their antinociceptive and anti-inflammatory properties. Mediators Inflamm. 2016. 1-9.

Parra, A., Martin-Fonseca, S., Rivas, F., Reyes-Zurita, F.J., Medina-O'Donnell, M., Martinez, A., Garcia-Granados, A., Lupiañez, J.A. and Albericio, F., 2014. Semi-synthesis of acylated triterpenes from olive-oil industry wastes for the development of anticancer and anti-HIV agents. European journal of medicinal chemistry, 74, pp.278-301.

Formisano, C., Rigano, D., Senatore, F. 2011. Chemical constituents and biological activities of *Nepeta* species. Chem Biodivers. 8: 1783-1818.

Fraga, B.M., Gonzalez-Coloma, A., Alegre-Gomez, S., Lopez-Rodríguez, M., Amador, L.J. Diaz, C.E. 2017. Bioactive constituents from transformed root cultures of *Nepeta teydea*. Phytochem. 133: 59-68.

Suntar, I., Nabavi, S.M., Barreca, D., Fischer, N., Efferth, T. 2017. Pharmacological and chemical features of *Nepeta L.* genus: Its importance as a therapeutic agent. Phytother Res. 32:185-198.

Sharma, A., Bhardwaj, G. and Cannoo, D.S., 2021a. Antioxidant potential, GC/MS and headspace GC/MS analysis of essential oils isolated from the roots, stems and aerial parts of Nepeta leucophylla. Biocatalysis and Agricultural Biotechnology, 32, p.101950.
